# Platelets and diseases: signal transduction and advances in targeted therapy

**DOI:** 10.1038/s41392-025-02198-8

**Published:** 2025-05-16

**Authors:** Yuchen Tian, Yao Zong, Yidan Pang, Zhikai Zheng, Yiyang Ma, Changqing Zhang, Junjie Gao

**Affiliations:** 1 Department of Orthopaedics, Shanghai Sixth People’s Hospital Affiliated to Shanghai Jiao Tong University School of Medicine, Shanghai, China; 2 Institute of Microsurgery on Extremities, and Department of Orthopedic Surgery, Shanghai Sixth People’s Hospital Affiliated to Shanghai Jiao Tong University School of Medicine, Shanghai, China; 3 Centre for Orthopaedic Research, Medical School, The University of Western Australia, Nedlands, Western Australia Australia

**Keywords:** Cardiology, Cardiovascular diseases, Inflammation, Cell biology, Physiology

## Abstract

Platelets are essential anucleate blood cells that play pivotal roles in hemostasis, tissue repair, and immune modulation. Originating from megakaryocytes in the bone marrow, platelets are small in size but possess a highly specialized structure that enables them to execute a wide range of physiological functions. The platelet cytoplasm is enriched with functional proteins, organelles, and granules that facilitate their activation and participation in tissue repair processes. Platelet membranes are densely populated with a variety of receptors, which, upon activation, initiate complex intracellular signaling cascades. These signaling pathways govern platelet activation, aggregation, and the release of bioactive molecules, including growth factors, cytokines, and chemokines. Through these mechanisms, platelets are integral to critical physiological processes such as thrombosis, wound healing, and immune surveillance. However, dysregulated platelet function can contribute to pathological conditions, including cancer metastasis, atherosclerosis, and chronic inflammation. Due to their central involvement in both normal physiology and disease, platelets have become prominent targets for therapeutic intervention. Current treatments primarily aim to modulate platelet signaling to prevent thrombosis in cardiovascular diseases or to reduce excessive platelet aggregation in other pathological conditions. Antiplatelet therapies are widely employed in clinical practice to mitigate clot formation in high-risk patients. As platelet biology continues to evolve, emerging therapeutic strategies focus on refining platelet modulation to enhance clinical outcomes and prevent complications associated with platelet dysfunction. This review explores the structure, signaling pathways, biological functions, and therapeutic potential of platelets, highlighting their roles in both physiological and pathological contexts.

## Introduction

Platelets, first found in 1842 and named in 1882, are important components of the blood that maintain blood circulation, stop bleeding, and repair tissues.^1^ These small, nucleus-free cells are generated from megakaryocytes (MKs) in the bone marrow and represent some of the smallest cellular structures in the human body.^2^ Despite their small size, platelets possess a highly complex structure, enriched with a variety of functional proteins and organelles that enable a broad range of biological functions. The cytoplasm of platelets contains various organelles, including mitochondria, lysosomes, and storage granules (such as alpha and dense granules), each contributing uniquely to platelet activation and function. Platelets also feature specialized canalicular systems, including the open canalicular system and the dense tubular system, which facilitate their interaction with other cells and structures within the circulatory system. Additionally, platelets are rich in cytokines and functional proteins, which contribute to various biological processes like platelet aggregation, wound healing, and immune modulation.^3–5^ The platelet membrane is covered with many different receptors and ligands, which enable platelets to bind to other cells and cytokines and fibrinogen on the damaged blood vessel wall, thereby promoting platelet aggregation and thrombus formation through different signal transductions,^6^ thereby participating in physiological processes such as thrombosis, immune response, and inflammation regulation.^7,8^

In addition to their primary roles in maintaining vascular function, hemostasis, and tissue repair, platelets are increasingly recognized for their involvement in the pathogenesis of various diseases, particularly cancer. They facilitate tumor growth and metastasis by releasing growth factors, promoting angiogenesis, and modulating immune responses. Additionally, platelets can protect tumor cells from immune system-mediated clearance, thereby promoting tumor immune escape.^9–12^

**Fig. 1:**
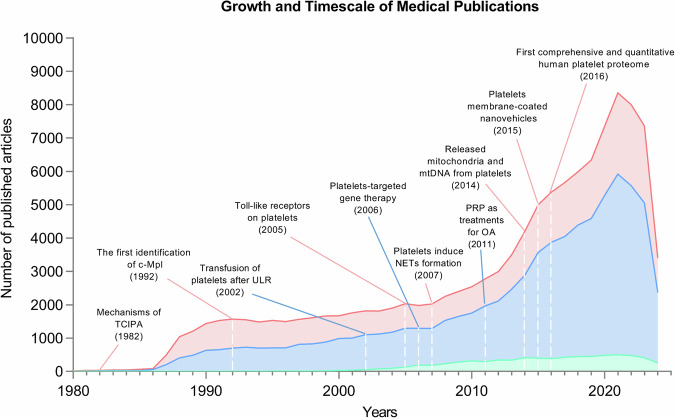
Number of growing published articles or studies from 1980 to Jun 2024, based on platelet medicine (pink), therapies (blue), and clinical trials (green). With the continuous discoveries of the structure and origin of platelets, as well as their involvement in many physiological and pathological precessions, the publication of using platelets for treatments has been increasing rapidly. The clinical trials studying platelet transfusion, PRP, and engineered platelets have also been increasing year by year. Data for this figure was extracted from PubMed by searching the term “platelet*” in combination with either “medicine,” “transfusion,” “PRP,” “therap*,” or “treatment.” Data of active clinical trials (recruiting, not yet recruiting, active, not recruiting, completed, enrolling by invitation, unknown status) were acquired from ClinicalTrials.gov

In the past five decades, thanks to research on platelet biology, platelets have been recognized as therapeutic targets in medicine and used as biologics for the treatment of multisystem diseases (Fig. 1). In clinical practice, platelets are key focal points for therapy, particularly in treating thrombotic disorders, inflammation, and tissue regeneration.^13^ Due to their central role in thrombosis, platelets have become key targets in antithrombotic therapy. By modulating platelet activation and aggregation, it is possible to effectively prevent conditions such as myocardial infarction and stroke, which are caused by abnormal clot formation. Commonly used antiplatelet drugs work by inhibiting platelet signaling pathways, thereby reducing the risk of thrombosis.^14^ Additionally, platelets are involved in immune regulation and inflammation, positioning them as promising targets for the treatment of chronic inflammatory diseases.^15^

In the review, we outline the structure and components of platelets, highlighting their multifaceted roles in maintaining vascular function, hemostasis, immune regulation and contributing to pathological conditions such as cancer. By understanding platelet biology in greater depth, including their signaling mechanisms and regulatory pathways, we can better appreciate their crucial roles in both health and disease. Furthermore, this knowledge provides a theoretical foundation for developing novel therapeutic strategies aimed at modulating platelet function in managing thrombotic diseases, inflammation, and cancer. The continued exploration of platelet biology is essential for the development of targeted therapies and for improving clinical outcomes in a wide range of diseases.

## The origin of platelets

The origin of platelets and whether platelets are living cells was highly controversial before the establishment of hematology.^1^ In 1921, Aldo Perroncito observed that platelets could replicate in peripheral blood.^16^ In 1923, Cesaris Demel reported that after stimulation of megakaryopoiesis, MKs migrate into blood vessels, where platelets are released from the surface of MKs. However, it was once believed that platelets originated from an undefined plasma component that precipitated after contacting with MKs,^17^ which did not match the accepted conclusion today that they originate from MKs.^18^

**Fig. 2:**
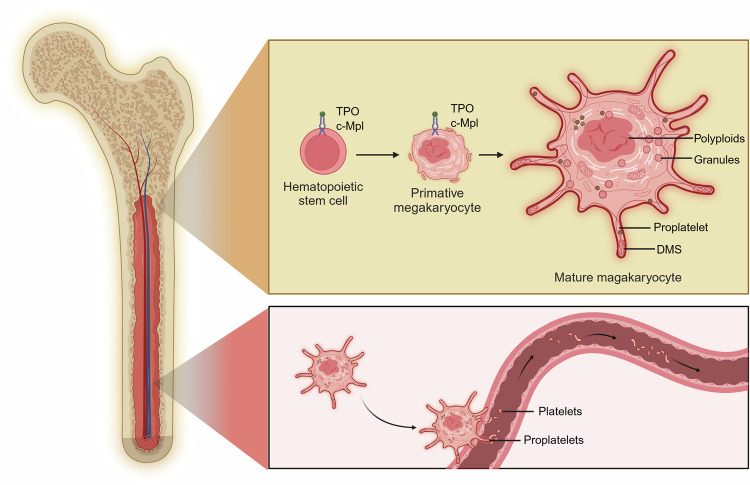
The origin of platelets. Platelets are mainly derived from MKs in the bone marrow. HSCs gradually differentiate into primitive MKs through continuous proliferation and differentiation into multipotent progenitors and downstream progenitors. Primitive MKs gradually mature, mediated by TPO, forming complex membrane systems and storage granules. Mature MKs form proplatelets, extend into the surrounding sinusoids, and separate platelets through membrane invagination and fusion

At first, multifunctional hematopoietic stem cells undergo directed differentiation in hematopoietic tissue to form primitive MKs.^19,20^ Some studies have also found that MKs exist in the yolk sac, fetal liver, and spleen during embryonic development, and regulate the migration of hematopoietic stem cells into niches.^21–23^ Besides, research has shown that lungs are the main site where late-stage platelets are produced, with many MKs circulating in the lungs and dynamically releasing platelets.^24^ During sepsis, the spleen is the main site for megakaryogenesis and platelet production.^25^ As development progresses, MKs form polyploids through endomitosis and are regulated by the SHP1/SHP2 and G6b-B, a receptor containing the immunoreceptor tyrosine-based inhibition motif (ITIM).^26,27^ The intracytoplasmic Golgi network forms a variety of storage granules through the budding of small vesicles containing abundant functional proteins.^28,29^ Cellular membranes are highly invaginated in dependence on actin fiber assembly, forming a demarcated membrane system.^30^ Eventually, primitive MKs become mature MKs.^31^ The differentiation, proliferation and maturation of MKs is mediated primarily by thrombopoietin (TPO) and the specific receptor c-Mpl.^32,33^ TPO binding to c-Mpl activates many downstream signaling pathways, such as the JAK2, PI3K/Akt, and MAPK/ERK1/ERK2 pathways.^34,35^ In addition, TPO promotes thrombosis, and c-Mpl receptors on the platelet surface regulate thrombosis by binding to circulating TPO.^36^ Mature MKs form long tubular extensions called proplatelets that extend into adjacent blood sinusoids, followed by invagination and fusion of the cell membrane surfaces, separating a portion of the MK cytoplasm.^37,38^ Finally, the MK cytoplasm surrounded by the membranes of these cells separates the components of MKs and enters the circulation as platelets through the sinusoidal blood vessels of the bone marrow hematopoietic tissue^39,40^ (Fig. 2). After release from MKs, platelets survive in the circulatory system for 7 to 10 days.^41^ Thus, sustained platelet production is essential to preserving normal platelet counts (i.e., 150–400 × 10^9^/L in adults).^42^

## Structure of platelets (Table 1)

**Fig. 3:**
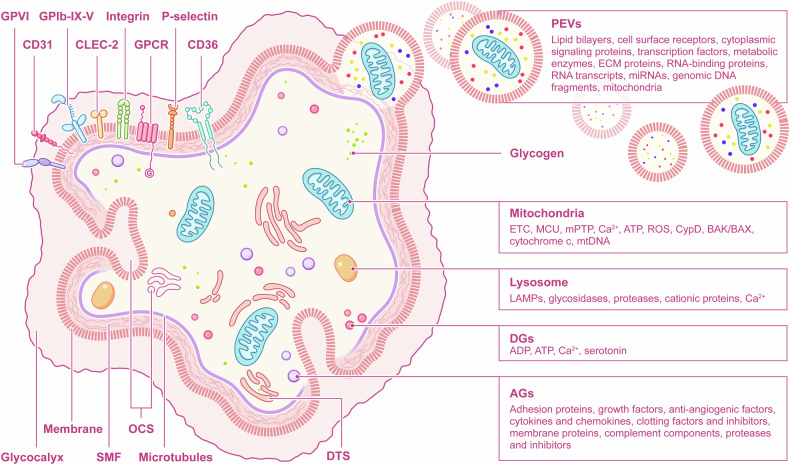
The structure and contents of platelets. Platelets participate in physiological and pathological processes through their complex structure and contents. There are multiple functional receptors expressed outside the platelet membrane and their glycan chain portions form surface glycocalyx. Platelets have no nuclei, and except for conventional organelles such as mitochondria and lysosomes, they also have unique tubular systems and storage granules. In addition, the platelet cytoskeleton provides support for platelet migration, adhesion and aggregation. Platelets also secrete vesicles that carry various signal factors, receptors, mitochondria, and nucleic acid

Platelets are non‐nucleated blood cells, in the form of discs that are slightly convex on both sides with a small diameter of 2–5 µm.^43^ Platelets have a typical double-layer membrane structure with abundant membrane proteins and contain mitochondria, lysosomal granules, glycogen granules, as well as store granules and other organelles. In addition, platelets have abundant and unique membrane structures.^44^ The various structures of platelets work in coordination to fulfill the important physiological functions of platelets (Fig. 3).

**Table 1:** Platelet structure, components, and corresponding functions

Structure	Components		Functions	Reference
Plasma membrane	Lipid bilayers		Containers of embedded cholesterol, glycolipids, and GPs and carriers of receptors.	^6^
	Contractile system		Consisting of actin filament and allowing platelets to change shape and translocate their receptors.	^49^
	Glycocalyx		Consisting of GPs and glycan chain portions. The site where receptors receive various signals and regulate most platelet functions.	^51^
	Ion channels		Maintaining intra- and extracellular ion concentration gradient and mediate platelet activation.	^122,123^
	Receptors	GPIIb/IIIa(α_IIb_β_3_)	Integrin family receptors. Adhering to fibrinogen, fibronectin, vitronectin and vWF to promote platelet aggregation.	^55^
		GPIb-IX-V	Leucine-rich glycoprotein family receptors. Receptors for vWF, fibrinogen and thrombin. Main platelet structural integrity and initial intracellular signaling to activate GPIIb/IIIa.	^60^
		P-selectin	Selectin family receptors. Specific marker of activated platelets and increasing the platelet-leukocyte interaction.	^67^
		TLRs	Recognizing PAMPs and DAMPs.	^74–76^
		P2Y_1_/P2Y_12_	ADP receptors. Participating in the ADP-induced platelet calcium inward flow during platelet aggregation and the inflammatory response process.	^85,88^
		PARs	Cleaved by thrombin and mediating hemostasis and thrombosis.	^95^
		PECAM-1/CD31	Immunoglobulin family receptors. Sites for the adhesion of monocytes and neutrophils and involved in the process of immune responses.	^101,102^
		ICAM-2/CD102	Immunoglobulin family receptors. LFA ligand and involved in platelet-leukocyte interaction.	^103^
		CLEC-2	Inducing platelet activation.	^104,105^
		GPIa-IIa	Collagen receptors.	^120^
		GPVI	Collagen receptors. Involved in the activation and formation of procoagulant platelets.	^111,112^
Mitochondria	ETC, MCU, mPTP, Ca^2+^, ATP, ROS, CypD, BAK/BAX, cytochrome c		Providing the energy for functions of platelets and mediating the activation and apoptosis of platelets.	^125,135,138,145,146,148^
	mtDNA		Involved in the activation of platelets and inflammation responses after release.	^151,155,161–163^
Lysosomes	LAMPs		Forming a protective layer against hydrolytic enzymes stored in the particles	^174,175^
	Glycosidases, proteases, cationic proteins		Involved in the degradation and recycling of intracellular materials, clearance of external substances, and regulation of normal function of platelets and the stability of the intracellular environment.	^176^
	Ca^2+^		Mediators of the activation of platelets.	^178,179^
DGs	ADP, ATP, Ca^2+^, serotonin		Playing an important role in platelet activation, platelet aggregation, vasoconstriction, and coagulation reactions.	^225–227^
AGs	Adhesion proteins, growth factors, anti-angiogenic factors, cytokines and chemokines, clotting factors and inhibitors, membrane proteins, complement components, proteases and inhibitors		Participating in various physiological and pathological processes like hemostasis, angiogenesis, platelet adhesion and aggregation, tissue repair and healing, fibroblast and endothelial cell migration, cell proliferation and differentiation, and immune regulation.	^211–213^
OCS	Lipid bilayers, contents within platelets		Playing an important role in material transportation, de-granulation, membrane expansion, and signal transduction.	^228,231–237^
DTS	Lipid bilayers, Ca^2+^, cAMPase		Key roles in calcium storage and release, signal transduction and lipid metabolism.	^238,239,242^
PEVs	Lipid bilayers, cell surface receptors, cytoplasmic signaling proteins, transcription factors, metabolic enzymes, ECM proteins, RNA-binding proteins, RNA transcripts, miRNAs, genomic DNA fragments, mitochondria		Key roles in coagulation, intercellular communication, inflammatory response, and angiogenesis.	^243,248,250–252^
Cytoskeleton	Microtubules, microfilaments, SMF		Playing a crucial role in maintaining platelet morphology, promoting platelet movement, regulating substance transport, and signal transduction.	^259,264–266,268^

### Platelet membrane and membrane receptors

Platelet biogenesis initiates during the maturation of MKs, when a pseudopod-like structure, referred to as a proplatelet, appears within the cytoplasmic lumen of the MKs.^45^ The demarcated membrane system, also called the invaginated membrane system, consists of a rich reservoir of cytoplasmic membranes and gives rise to both proplatelet and platelet membranes.^46,47^ The platelet plasma membrane is composed of lipid bilayers containing embedded cholesterol, glycolipids, and glycoproteins (GPs) serving as carriers for platelet adhesion, activation, and aggregation through receptors.^6^ In addition, negatively charged phosphatidylserine (PS) is expressed by activated platelet membranes. PS transfers inside-out and helps to generate thrombin during the coagulation propagation stage.^48^ The submembrane region enables platelets to undergo shape changes and receptor relocation.^49,50^

#### Platelet membrane receptors and downstream signal transduction

The glycocalyx, a cellular coat of the platelet, consists of various GPs and their glycan chain portions and is the site where platelet receptors are located. It contacts with the cellular microenvironment and is the most energized part of the platelet.^51^ Platelet membranes are enriched with specific surface receptors that precisely regulate signal-dependent platelet activation and modulate granule release, adapting to processes such as coagulation, inflammation, anti-microbial defense, angiogenesis, wound healing, and metastasis.^44,52^

##### Integrins α_IIb_β_3_

The integrins are a family of transmembrane GP signaling receptors mediating interactions and are composed of two evolutionarily conserved type I transmembrane GPs, α and β.^53^ The integrin family can be divided based on the β subunit. β_1_ and β_3_ are present in platelets and form five platelet integrins with different α subunits, including α_2_β_1_, α_5_β_1_, α_6_β_1_, α_v_β_3_, and α_IIb_β_3_.^54^ α_IIb_β_3_ (GPIIb/IIIa), the predominant integrin on platelets, is essential for platelet aggregation and can attach to many ligands such as fibrinogen, fibronectin, vitronectin, von Willebrand factor (vWF), and other adhesion protein molecules.^55,56^ The activation of α_IIb_β_3_ is regulated via inside-out signaling, in which agonists (ADP or thrombin) interact with G protein-coupled receptors (GPCRs), or adhesion proteins (collagen or vWF) bind to GPIb-IX-V or GPVI. This triggers intracellular signaling that induces a conformational shift in the integrin, increasing the affinity of extracellular structural domains of α_IIb_β_3_ for ligands, which can be inactivated by dissociation of calcium chelators such as ethylene diamine tetraacetic acid (EDTA).^57^ Soluble fibrinogen triggers the outside-in signaling of integrin α_IIb_β_3_, and activated α_IIb_β_3_ triggers the activation of protein kinase C (PKC) δ, mediating the irreversible stable adhesion, migration, cytoskeleton rearrangement, and aggregation of platelets, as well as subsequent thrombosis.^58^ This process is regulated by proline-rich tyrosine kinase 2 (Pyk2).^59^

##### GPIb-IX-V

GPIb-IX-V complex, a leucine-rich glycoprotein family receptor, is involved in cell signaling, adhesion, growth and development.^60^ GPIb-IX-V consists of four transmembrane proteins. GPIb forms a 1:1 complex with GPIX, and GPV is expressed in the form of a loosely bound complex with GPIb-IX.^61^ The functions of GPIb-IX-V include vWF receptor, thrombin receptor, maintenance of platelet structural integrity, platelet attachment to endothelial cells, and recruitment of leukocytes to the site of injured vessels.^62^ Under high shear rate flow conditions, its interaction with vWF on the subendothelial matrix promotes platelet adhesion at the site of injured vessels, leading to platelet arrest and is critical for initial hemostasis.^7^ The binding of GPIb-IX cytoplasmic domain to 14-3-3 protein plays a role in signal transduction to regulate the extracellular ligand binding activity of GPIb-IX.^63^ Besides, Src family kinases (SFKs), especially Lyn, regulate the intracellular signaling pathway of GPIb-IX. Lyn-dependent phosphorylation and activation of guanine nucleotide exchange factors (GEF) activate Rac1, a small GTPase member of the Rho family,^64^ activating the PI3K pathway. PI3K activates the Akt/cGMP/p38MAPK signaling pathway, further leading to the activation of GPIb-IX-mediated ligand binding function of integrin α_IIb_β_3_.^65^ In addition, GPIb-IX-V is crucial for platelet survival in circulation and plays a significant role in platelet clearance.^66^

##### P-selectin

The selectin family is calcium dependent cell adhesion molecules including E-, L-, and P-selectin.^67^ P-selectin (CD62) presents on the surface of activated endothelial cells and platelets.^68^ The P-selectin of platelets is generally stored in α granules. When platelets are activated, P-selectin can be quickly transferred outside, which is considered to be a specific marker of activated platelets, increasing the interaction of endothelial cells and platelets and leukocytes.^67^ The expression of P-selectin is regulated by multiple signaling pathways, such as protein tyrosine phosphorylation, Na^+^/H^+^ exchange, and Ca^2+^ mobilization.^69^ In addition, the proteolysis of P-selectin on the platelet membrane contributes to the soluble P-selectin in the circulation.^70^

##### Toll-like receptors (TLRs)

TLRs are a family of pattern recognition receptors (PRRs). They are expressed on platelets and can recognize pathogen-associated molecular patterns (PAMPs) and host-derived DAMPs. For example, TLR4 can recognize lipopolysaccharides (LPS) of Gram negative bacteria and DAMPs, while TLR2 can recognize peptidoglycans of Gram positive bacteria.^71^ TLR7 signaling plays a critical role in platelet activation and PLA formation during bacterial sepsis,^72^ and in platelet-neutrophil aggregate formation in lupus nephritis.^73^ When TLRs bind to PAMPs or DAMPs, the activation of TLR leads to the activation of downstream NF-κB and MAPKs signaling pathways through both MyD88 dependent and independent signaling, thereby promoting platelet activation and the production and release of immunomodulatory factors like RANTES, CD40L, PF4, and P-selectin.^74–76^

##### G protein-coupled receptors (GPCRs)

GPCRs are a family of cell surface receptors,^77^ which trigger the activation of the intracellular second messenger signaling pathway by binding to guanine nucleotide-binding protein (G protein).^78^ Without ligands, the Gα subunit binds to GDP and forms a heterotrimer with Gβγ, interacting with the cytoplasmic ring of GPCRs. Gβγ promotes the binding of Gα and GPCR receptors and serves as a guanine nucleoside dissociation inhibitor (GDI) for Gα-GDP, slowing down the exchange of GDP and GTP. The ligand-bound GPCR functions as a guanine nucleotide exchange factor (GEF), inducing conformational changes in the Gα subunit to enable it to exchange GTP for GDP. Gβγ and Gα-GTP separate and emit signals to their respective effectors.^79^ Currently, three types of G proteins have been identified as the main mediators of platelet activation: Gα_q_, Gα_i_, and Gα_13_.^80–82^ However, even without Gα_q_, Gα_i_, or Gα_13_, platelet can also be activated, although higher ligand concentrations are required than wild-type platelets.^83^ Therefore, platelet activation via GPCRs is a complex process that engages multiple G protein signaling pathways. Many mediators of platelet activation contact with GPCRs, including ADP receptors, protease-activated receptors (PARs), thromboxane A_2_ (TXA_2_) receptors, adrenergic receptors, and prostaglandin receptors.^9^

**Fig. 4:**
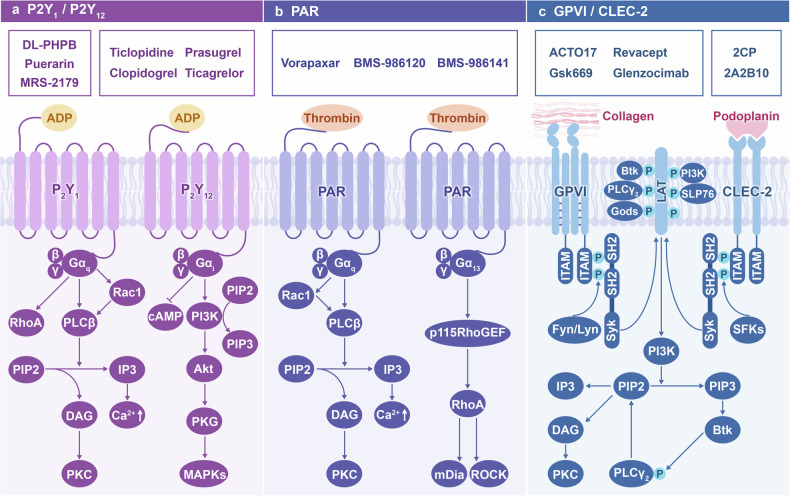
Signal transduction and targeted drugs of **a** P2Y_1_/P2Y_12_, **b** PARs, and **c** GPIV/CLEC-2. P2Y_1_/P2Y_12_ and PARs are important GPCRs for platelets, which recognize ADP and thrombin, respectively, and trigger downstream signal transduction through coupled different G proteins, including PLCβ, Rho-GEF, PI3K, etc. GPVI and CLEC-2 are platelet surface immunoglobulin family receptors that participate in platelet activation and immune response through ITAM. These receptors and signal transduction collectively promote activation reactions mediated by platelet calcium influx, including secretion of granules, aggregation, thrombosis, etc. Targeted drugs targeting different receptors can effectively inhibit platelet activation, thereby reducing thrombus formation

ADP induced platelet activation requires co stimulation of Gα_q_-coupled P2Y_1_ receptors and Gα_i_-coupled P2Y_12_ receptors,^84^ which triggers calcium inward flow and induces platelet aggregation and secretion during hemostasis.^85^ The stimulation of Gα_q_ signaling downstream of P2Y_1_ receptor activates phospholipase C (PLC) β. PLCβ hydrolyzes membrane phosphatidylinositol diphosphate into inositol triphosphate (IP3) and diacylglycerol (DAG),^86^ resulting in increased cytoplasmic Ca^2+^ concentration and PKC activation, increased platelet secretion, and activation of α_IIb_β_3_-mediated signaling, triggering a series of intracellular events.^87^ Besides, P2Y_1_ is involved in the inflammatory response process through stimulating platelets through Rho-GTPase signaling.^88^ Rho family GTPases are small signal G proteins of the Ras superfamily that circulate between inactive GDP-bound forms and active GTP-bound forms under the regulation of regulatory factors to regulate various cellular processes. For example, RhoA promotes platelet shape changes, granule release, diffusion, and thrombus retraction through Rho-associated coiled-coil containing protein kinase (ROCK) and mammalian diabetic homolog (mDia).^89^ Besides, Rac1 activates platelets by stimulating PLC and calcium mobilization.^90^ On the other hand, stimulating Gα_i_-coupled P2Y_12_ receptors can inhibit cyclic adenosine monophosphate (cAMP) production and activate PI3K, which is crucial for sustained activation of integrin α_IIb_β_3_.^91^ The activation of Gα_i_ signaling downstream of P2Y_12_ requires the involvement of lipid rafts, which is crucial for its role in ADP-mediated platelet activation^92^ (Fig. 4a).

TXA_2_ receptor coupled with Gα_q_ and Gα_12/13_.^93^ cAMP-dependent kinase mediates Gα_13_ phosphorylation and preferentially inhibits TXA_2_-mediated signaling in platelets.^94^ The lack of Gα_13_ significantly reduces the efficacy of TXA_2_ in inducing platelet activation in vitro. These defects are along with a decrease in RhoA activation, making it impossible to generate stable platelet aggregates under high shear stress in vitro.^82^

PARs are a group of membrane proteins that undergo cleavage by proteases, especially thrombin, exposing bound ligands to initiate platelet activation. As pivotal regulators of platelet function in hemostasis and thrombosis, they represent valuable targets for antiplatelet therapies.^95^ PARs are functionally coupled with Gα_q_, Gα_12/13_, and in some cases, Gα_i_.^96^ After thrombin stimulation, the Gα_13_ subunit activates p115RhoGEF, followed by RhoA activation.^97^ Platelets stimulated by thrombin generate extracellular ROS by activating the signaling pathway that binds PAR4 and GPIbα, further amplifying the initial signal and maintaining platelet recruitment and activation^98^ (Fig. 4b).

##### Immunoglobulin receptors

Immunoglobulin family receptors, found on almost all blood cells, including platelets, feature at least one subunit containing an extracellular immunoglobulin superfamily domain and/or an intracellular immune receptor tyrosine activating motif (ITAM) or inhibitory motif (ITIM). These receptors are primarily involved in mediating immune responses.

Human platelets express six members of the FcγRs, which are unique immunoglobulin G antibody receptors that activate signals and cells through ITAM transduction, except for FcγRIIB.^99^ Among them, FcγRIIA is most abundantly expressed in the circulation and activates platelets by recognizing and responding to circulating immune complexes (ICs).^100^

The platelet endothelial cell adhesion molecule (PECAM-1)/CD31, which provides sites for the attachment of monocytes and neutrophils, highly enriched at cell-cell junctions, participates in the process of inflammation, trauma healing^101^ and the entry of a number of viruses into the platelet (e.g., human defective virus, poliovirus).^102^

Intercellular adhesion molecule-2 (ICAM-2)/CD102, which is present on the membrane surface and in the open canalicular system (OCS), is the only known platelet surface lymphocyte function-associated antigen (LFA) ligand that is involved in platelet-LFA correlation and is crucial for platelet-leukocyte communication.^103^

C-type lectin-like receptor (CLEC-2) induces platelet function through a pathway that is dependent on the tyrosine kinases in CLEC-2 and on the hem-ITAM pathways to induce platelet activation.^104,105^ After binding to its ligand, CLEC-2 can be phosphorylated by platelet tyrosine kinase,^106^ and phosphorylated hem-ITAM activates spleen tyrosine kinase (Syk) through the P13 kinase/Bruton’s tyrosine kinase (Btk) pathway.^107^ Besides, SFKs bind to phosphorylated hem-ITAM and lead to the activation of SFKs, further enhancing the activation of Syk.^108^ Activated Syk and SFKs phosphorylate LAT, forming the LAT signaling bodies. There are many tyrosine residues on LAT and can be phosphorylated by protein kinases. Thus the LAT serves as a site for proteins containing SH2 domains, forming multiprotein complexes.^109^ PI3K activation in the LAT signaling bodies results in the production of phosphoinositol-3,4,5-triphosphate (PIP3), activating Btk and phosphorylates PLCγ_2_. PLCγ_2_ hydrolyzes PIP2 into IP3 and DAG, mediating the release of intracellular stored Ca^2+^ and the influx of extracellular Ca^2+^ ^110^ (Fig. 4c).

GPVI, the receptor of collagen expressed exclusively in platelets and mature MKs,^111^ guides the activity of procoagulant platelets via binding to collagen, and the binding of GPVI and fibrin greatly promotes the activation of procoagulant platelets.^112^ GPVI and FcRγ chain are expressed in a non-covalent manner, and their coupling mediates platelet activation through the phosphorylation of conserved ITAM tyrosine residues on FcRγ chain induced by Fyn, and Lyn.^113^ Subsequently, Syk was recruited to phosphorylated ITAM through the SH2 domain and activated, initiating downstream signal transduction, resulting in phosphorylation and activation of PI3K and effectors such as LAT, SLP-76, Gads, and jointly promoting phosphorylation and activation of PLCγ_2_ and PKC.^114^ The PI3K-Akt pathway is an essential signaling pathway for the downstream activation of GPVI triggered by collagen, mainly mediating GPVI-dependent calcium influx through α and β subunits.^115^ The adapter protein SLP-76 is a key regulator that exists between Syk and PLCγ_2_ in the GPVI-mediated activation signaling cascade, and the complete GPVI/FcRγ/SLP-76 signaling pathway participates in collagen-mediated procoagulant activity of platelets.^116^ The PKCε and PKCθ promote platelet diffusion, secretion, and aggregation during collagen-activating GPVI.^117,118^ In addition, phospholipase A_2_ (PLA_2_) activation is critical for the production of TXA_2_ following GPVI stimulation^119^ (Fig. 4c).

##### Other receptors

There are also other receptors on the platelet surface, including collagen receptors GPIa-IIa (thrombospondin, TSP),^120^ C1q receptor, as well as coagulation and fibrinolytic protein receptors, 5-HT receptor, and the membrane protein GPIV/CD36.^121^

#### Ion channels

The platelet membrane contains calcium channels, sodium pumps, and anion pumps that regulate the ion concentration gradient across the cell. Shear stress activates mechanosensitive Ca^2+^ channels, prompting an influx of Ca^2+^. This elevation in intracellular Ca^2+^ activates TMEM16F, which mediates PS exposure, stimulates purinergic signaling and boosts calpain activity. Calpain then cleaves actin cytoskeletal proteins, such as talin, in activated platelets, facilitating enhanced α_IIb_β_3_ integrin activation on the platelet surface.^122,123^

### Mitochondria

Each platelet contains 5 to 8 mitochondria, which share the same structure as those found in other cells. These mitochondria are characterized by two concentric membranes—the outer and inner membranes—with the inner membrane forming invaginations into the mitochondrial matrix.^124^

#### Mitochondria regulate energy metabolism in platelets

Mitochondria are the most important source of energy for platelets. In resting platelets, 60% of the energy is derived from glycolysis and 40% from oxidative phosphorylation (OXPHOS).^125^ The processes of platelet activation, diffusion between damaged blood vessels or fibrin, and clot retraction all require much energy. Normal expression of platelet mitochondrial genes is necessary for fibrinolysis, hemostasis, and coagulation in response to injury.^126,127^

Research has shown that resting platelets can seamlessly transition between glycolysis and oxidative phosphorylation (OXPHOS), utilizing either glucose or fatty acids as fuel sources. When activated by thrombin, platelets rapidly uptake exogenous glucose through glucose transporter 3 (GLUT3).^128^ As platelets predominantly convert glucose to lactate rather than utilizing it in the mitochondrial tricarboxylic acid cycle, most of the mitochondrial ATP required for granule secretion and thrombus formation is derived from fatty acid oxidation.^129^ When the utilization of glutamine, fatty acids, and glucose is inhibited, platelets compensate by increasing the flux of glycolysis.^130^ Mitochondria split through Drp1-dependent pathway and transit to a glycolytic phenotype.^131^ Mitochondrial enzyme pyruvate dehydrogenase kinases phosphorylate pyruvate dehydrogenase complexes to inhibit their activity, thereby transferring pyruvate flow from OXPHOS to aerobic glycolysis during platelet activation, enabling platelets to respond to various conditions, such as hypoxia or the presence of mitochondrial inhibitors.^132^ Therefore, simultaneously inhibiting aerobic glycolysis and pentose phosphate pathways can effectively suppress agonist-induced platelet responses.

#### Mitochondria regulate platelet activation

Increasing evidence suggests that mitochondria in platelets regulate prothrombotic functions of platelets not only through energy production but also through redox signaling, oxidative stress, and the maintenance of calcium homeostasis.^133^ When platelets adhere to the vascular endothelium, several agonists activate circulating platelets and recruit them to the thrombus. The concentration of intracytoplasmic Ca^2+^ increases and enters the mitochondria via the voltage-dependent anion-selective channels (VDAC) and the mitochondrial Ca^2+^ uniporter (MCU). As a second messenger, Ca^2+^ plays a role in regulating cytoskeleton reorganization, activation of GPIIb/IIIa, granule and vesicle release, aggregation, and thrombus formation.^134^ Then, the mitochondrial ΔѰ_m_ and OXPHOS increase rapidly and transiently,^135^ and the mitochondrial membrane potential is hyperpolarized, with an increase in ATP and reactive oxygen species (ROS) production, acting as second messengers that regulate several signaling pathways.^136^ The hyperpolarization of the mitochondrial membrane leads to electron leakage from the electron transport chain (ETC), subsequently promoting production and release of mitochondrial O^2−^ through ETC complexes I and III, and converting it to H_2_O_2_ through SOD2.^137^ Increased endogenous ROS production, in turn, alters mitochondrial function and promotes the initiation of the PS exposure essential for platelet adhesion and activation in circulation.^138^ Through a positive feedback mechanism, ROS transport between mitochondria leads to an increase in ROS production, accompanied by ROS burst and release leading to the collapse of ΔѰ_m_ and the formation of mitochondrial permeability transition pores (mPTP) in a calcium independent manner.^139^

#### Mitochondria regulate platelet apoptosis

Hyperactivation also triggers excessive thrombosis and thromboembolic complications, as well as apoptosis in platelets leading to thrombocytopenia and bleeding.^140,141^ This pathway is initiated by a strong stimulus, with increased cytoplasmic and mitochondrial calcium levels leading to an increase in ROS,^142^ depolarization of the mitochondrial membrane potential, and opening of the cyclophilin D (CypD)-dependent mPTP.^143^ MPTP is a nonselective multiprotein pore that crosses the inner and outer mitochondrial membranes, leading to a rapid collapse of ΔΨm due to impaired proton transfer to the mitochondrial intermembrane space.^144^ This process is accompanied by intra-mitochondrial ROS production that further promotes circulating oxidative stress, creating a vicious cycle. Simultaneously, apoptotic proteins BAK/BAX translocate to the mitochondria causing irreversible mitochondrial injury through mitochondrial outer-membrane permeabilization (MOMP),^145,146^ and cytochrome c is released from the mitochondrial matrix to the cytoplasm, initiating a signaling cascade that promotes apoptotic caspase activation.^147^ Together, the two pathways lead to PS exposure and induction of platelet apoptosis.^148^ In addition, platelets inherently undergo cell death, and the key proteins Bcl-x_L_, BAK, and BAX, pivotal elements of the mitochondrial apoptosis pathway, are central to the molecular clock that regulates platelet lifespan. With aging, the degradation of Bcl-x_L_ triggers BAK-mediated platelet apoptosis, resulting in their removal from circulation.^149,150^

#### Released mitochondria and mitochondrial DNA (mtDNA)

Activated platelets also release mitochondria and mtDNA. It was first revealed by Boudreau that platelets release mitochondria both within microparticles and freely. Mitochondria released by platelets are hydrolyzed by bactericidal secreted PLA_2_ IIA (sPLA_2_-IIA), leading to the release of pro-inflammatory lipid mediators and mtDNA, and promoting neutrophil proinflammatory responses.^151^ Platelet-released mitochondria are a major source of circulating mitochondria and interact with multiple cells, fulfilling a wide range of biological functions,^152–154^ and are often considered potential damage-associated molecular patterns (DAMPs) sources that promote inflammation and oxidative stress in diverse human diseases.^155,156^ Further research on platelet released mitochondria has shown that mitochondria from platelets can be captured by tumor cells, myocardial cells, nerve cells and mesenchymal stem cells (MSCs), as functional mitochondrial sources that promote tumor metastasis,^157^ angiogenesis,^154^ improve cardiac function,^158^ cognitive impairment,^159^ and treat ischemia-reperfusion injury.^160^ The mtDNA derived from platelets can induce platelet activation and, with proinflammatory properties, leads to the initiation of inflammatory responses.^161–163^

#### Biomarkers of multiple diseases

Beyond their role in regulating hemostasis, platelet mitochondrial function has long been utilized as a model for studying mitochondrial dysfunction in human diseases, as platelets are more accessible than other metabolically active tissues.^137^ Studies have shown that platelet mitochondria exhibit significant functional alterations in central nervous system (CNS) disorders like stroke, subarachnoid hemorrhage, CNS infections, and psychiatric disorders.^164–168^ Assessment of platelet mitochondrial function contributes to the diagnosis and severity assessment of these diseases.^169^ In addition, the mtDNA in circulation mainly reflects the copy number of mtDNA in leukocytes and platelets. Therefore, quantification of mtDNA in peripheral blood can also indirectly reflect platelet mitochondrial function and serve as a biomarker for various diseases.^170,171^

### Lysosomes

There are three main storage granules, alpha granules, dense granules, and lysosomes in platelets, which carry different cargoes and have different mechanisms of biogenesis, transport, and exocytosis.^6,172,173^ Lysosomes have highly glycosylated membrane proteins, such as the lysosome-associated membrane proteins (LAMPs). They create a protective barrier against hydrolytic enzymes stored within the particles.^174,175^ Hydrolases in lysosomes are used to activate platelets captured in the formation of clots and may ultimately be used to remodel the site of injury.^176^ Lysosomes release proteins through cytosolization, depending on the general fusion proteins N-ethylmaleimide-sensitive factor and SNAP-23 and requires two different heterodimers (SNAP-23/syntaxin2 and SNAP-23/syntaxin4).^177^ In addition, the lumen of lysosomes is enriched with calcium ions.^178,179^

### Alpha granules (AGs)

One of the main functions of platelets is to secrete various proteins that can regulate thrombus formation, promote wound repair, and aid in cell adhesion. Most of these secreted proteins are stored in AGs.^180^ AGs are unique and most abundant in platelets, with a diameter of 0.2–0.4 μm. A single platelet may contain 50 to 80 AGs.^181–183^

A variety of proteins are synthesized in the rough endoplasmic reticulum of megakaryocytes (MKs) and are subsequently packaged into storage vesicles by the Golgi apparatus, forming the contents of the alpha granules (AGs). Other proteins are acquired through liquid-phase endocytosis.^184^ Proteomic analysis of AGs validated 284 proteins, 50 of which showed 65% overlap with 81 proteins released by platelets.^185^ During platelet activation, AGs secrete multiple bioactive molecules involved in key cellular functions like adhesion proteins,^186,187^ growth factors,^188,189^ cytokines and chemokines,^190–195^ clotting factors and inhibitors,^196–200^ membrane proteins,^56,201–205^ complement components,^206^ and proteases and protease inhibitors^207–214^ (Table 2). Most membrane proteins present within AGs are expressed on resting platelets.^215^ However, the membranes of alpha granules (AGs) contain specific molecular receptors, such as P-selectin, TREM-like transcript-1, CD40L, and GMP-33, with ligand-recognition sites facing the internal side, which become exposed only upon platelet activation and do not present in the plasma membrane of resting platelets, which can be identified as biomarkers of platelet activation.^211^

**Table 2:** Functional proteins released by AGs after platelet activation

Proteins		Functions	Reference
Adhesion proteins	vWF, fibronectin, vitronectin	Mediators of platelet-platelet and platelet-endothelium adhesion and interaction in hemostasis and thrombosis.	^186,187^
Growth factors	PDGF, TGF-β, VEGF, IGF, EGF, FGF, ANG-1, CTGF, HGF	Roles in the promotion of cell migration, regulating cell proliferation and differentiation, promoting angiogenesis, improving blood supply, and assisting in tissue repair.	^188,189^
Anti-angiogenic factors	Angiostatin, endostatin	Inhibitors of angiogenesis.	^531,532^
	TSP-1	Inhibitors of endothelial cell proliferation and stimulating endothelial cell apoptosis.	^533^
Cytokines and chemokines	PF4(CXCL4)	Binding with heparin to participate in heparin-induced thrombocytopenia (HIT). Multiple effects in platelet coagulation interference, inflammatory response, vascular inhibition, and anti-tumor properties.	^190,191^
	IL-1	Promoting platelet aggregation, vascular occlusion, endothelial permeability, and cascade inflammatory response.	^192^
	TNF	Promoting inflammation and coagulation, and downregulating the thrombomodulin-Protein C anticoagulant pathway.	^193,194^
	CXCL1/5/7/8/12, CCL2/3/5	Affecting the counts of circulating white blood cells, leukocyte activation and recruitment, platelet activation and coagulation system. Participating in thromboinflammation and immune thrombosis formation.	^195^
Clotting factors and inhibitors	Fibrinogen	Promoting fibrin formation and helping stop bleeding.	^196,197^
	Plasminogen	Participating in the plasminogen activation system (PAS) and hydrolyze the fibrin.	^534^
	FII, FV, FVIII, FXI, FXIII	Participating in coagulation.	^198^
	Plasminogen activator inhibitor-1 (PAI-1), antiplasmin	Restricting fibrinolysis mediated by plasmin.	^535^
	Antithrombin	Cutting coagulation factors to inhibit coagulation.	^536^
	Nexin-2, protein S, tissue factor pathway inhibitor (TFPI)	Inhibitors of coagulation factors.	^199,200^
Membrane proteins	CD40L	Promoting the expression of a wide range of atherogenic mediators. Important mediator of leukocyte immune responses and recruitment.	^201^
	GPIIb/IIIa	Receptors of various adhesion proteins to mediate platelet aggregation.	^56^
	GPIb-IX-V	Main receptors of vWF, fibrinogen and thrombin to mediate platelet adhesion and aggregation.	^202^
	GPVI	Receptors of collagen to mediate ITAM-dependent signaling and platelet activation.	^203^
	P-selectin	Transported to the plasma membrane and interacting with other cell receptors as a cell adhesion receptor after platelet activation. Participating in the interaction between platelets and endothelial cells, monocytes, neutrophils, and lymphocytes.	^214^
	TLT-1	Supporting platelet aggregation during vascular injury and participating in inflammatory diseases and sepsis.	^204,205^
	PECAM-1/CD31	Roles in cell adhesion, signal transduction, calcium modulation, cell apoptosis, angiogenesis, thrombosis and inflammation.	^101,537^
Complement components	C1, C3, C4	Participating in complement activation cascade.	^206^
Proteases and protease inhibitors	Heparanase	Roles in the degradation of heparan sulfate chains and regulation of coagulation process.	^207^
	C1 inhibitor	Roles in the degradation of FXIa, FXIIa, and plasma kallikrein.	^208^
	Antitrypsin	Protecting tissues from the effects of human neutrophil elastase (HNE) and other proteases released by neutrophils in an inflammatory state.	^538^
	Antichymotrypsin	Involved in acute phase reactions, inflammation, and protein hydrolysis.	^539^
	Matrix metalloproteinase (MMP) and tissue inhibitors of metalloproteinases (TIMP)	Mediators of adhision and aggregation of platelets, angiogenesis, inflammation and tumor metastasis.	^209,210^

Platelet activation induces secretion of AGs and release of their cargo via cytokinesis. Secretion of AGs is crucial for coagulation, but excessive release promotes occlusive thrombosis. The secretion of AGs is affected by a variety of receptors and signaling pathways.^183,216^ Soluble NSF attachment protein receptors (SNAREs) are present at both granule and plasma membranes and mediate the exocytosis of platelet AGs by binding to each other to form four helical bundles that drive the fusion of the granule and cell membranes.^217,218^ Key SNAREs include VAMP-8, syntaxin-2, and SNAP-23.^219,220^ Sec/Monc18-like (SM) proteins act as initiator of SNAREs to assemble into tight SNARE complexes that fuse and release platelet AGs.^221^ In addition, the septins (Sept), a widely expressed protein family, is a unique component of the cytoskeleton, and deletion of Sept8 inhibits the exocytosis of AGs.^222^

### Dense granules (DGs)

DGs are small storage granules unique to platelets, containing calcium, adenosine monophosphate, and serotonin, crucial for the activation of platelets.^223^ DGs are the tiniest granules in platelets, with an average diameter of 150 nm.^181,224,225^

DGs contain smaller and simpler molecules than AGs and contain a high concentration of adenine nucleotides and an ADP/ATP ratio of 1.5, the opposite of the ratio found in the whole platelets.^126^ They also store the majority of the total platelet content of divalent cations, with calcium being the predominant ion in human platelets, more than 100 times that of the whole platelet.^226,227^ Upon platelet adhesion to the damaged vascular endothelium, dense granules (DGs) release various activators, including ADP, to initiate platelet activation. Simultaneously, signaling pathways involved in platelet activation induce the release of Ca2+ from the DGs. As the concentration of Ca2+ rises, the levels of cyclic adenosine monophosphate (cAMP) decrease, triggering the activation of protein kinases such as Syk and PKC. This cascade leads to the discharge of DG contents, including ADP and serotonin, which further enhance platelet activation, aggregation, and contribute to the hemostatic process.^225^

### Open canalicular system (OCS)

Platelets have a unique membrane structure and tubular system. The OCS is a complex intracellular network of membrane channels connected to the plasma membrane, and its main function is to transport substances into and out of platelets.^228–230^

OCS plays a role in platelet activation. On one hand, OCS functions as a membrane reserve, facilitating the expansion necessary for platelet shape alteration and spreading, while also acting as a storage location for platelet membrane receptors.^228,231–233^ On the other hand, OCS transport contents stored within platelets to the external environment.^234^ Upon platelet activation, AGs and DGs combine with the OCS or plasma membrane, discharging their contents and amplifying platelet activation.^235^ More recently, it has been proposed that OCS also contributes to the regulation of platelet calcium signaling.^236^ It has been observed that calcium signaling in activated platelets is initiated and spread in distinct areas of the platelets, which aligns with the widespread distribution and surface-associated characteristics of the OCS.^237^

### Dense tubular system (DTS)

The DTS is an internal smooth endoplasmic reticulum membrane system that participates in initiating and regulating platelet activation. The DTS is thinner than OCS, without continuity with the plasma membrane.^238^ DTS contributes to the regulation of the platelet release response because it stores two important regulators of platelet activation, the calcium pool, and the adenyl cyclase (cAMPase).^239^ DTS stores 30% of total platelet calcium content. Calcium mobilization involves its release, related with the activation of PLC and PKC, producing IP3-dependent signals that act on the IP3 receptor in DTS,^240^ into the cytoplasm where it activates many calcium-dependent enzymes (phospholipase A2, myosin light chain kinase, proteases including calpain).^241^ When calcium levels in the DTS drop, stromal interaction molecule 1 binds to the calcium release-activated calcium channel regulator 1, allowing extracellular Ca^2+^ to enter the cytoplasm. This influx triggers platelet activation, initiating Ca^2+^-dependent signaling pathways, metabolic processes, and enhancing degranulation and PS exposure on the platelet surface.^242^ In addition, DTS is a major site of prostaglandin biosynthesis. It stores phospholipid-modifying enzymes that catabolize arachidonic acid to TPO.^238^

### Platelet-derived extracellular vesicles (PEVs)

Extracellular vesicles (EVs) include micromembrane vesicles, microvesicles, and exosomes.^243,244^ Platelets also synthesize and secrete EVs. PEVs are important cellular components in the blood circulation, participating in inflammation and immune responses through interaction with inflammatory and immune cells.^245^ PEVs are divided into two main types: exosomes with a diameter of about 40 to 100 nm, and platelet-derived microvesicles (PMVs) with a diameter of 100 to 1000 nm, expressing GPIIb/IIIa, GPIbα, and P-selectin.^246,247^ Activation of platelets by agonists and Ca^2+^ ionic peptides increases intracellular calcium ions, resulting in immediate exposure of PS. Subsequently, cytoskeletal proteolysis is triggered, leading to membrane cleavage and release of PMVs.^248^ PMVs are the most abundant microvesicles in the blood.^249^ PMVs carry different proteins that play a key role in cellular communication and responses, triggering the release of cytokines that are involved in inflammation, cancer progression, angiogenesis, metastasis, and tissue repair.^243,250–252^ In addition, PMVs have been reported to carry mitochondria.^151,152^ It is worth noting that the dynamic changes of PMV contents mainly depend on the platelet activation mechanism, the agonists used, and the stimulation time. Various activation pathways can result in the generation of heterogeneous PMV populations, each with distinct surface marker profiles and protein mass spectrometric signatures, potentially influencing their functions in intercellular communication.^253^ PMVs can affect the microenvironment and cells by (1) triggering cell surface receptors, (2) translocation of receptors to the cell surface, or (3) direct transfer of mRNAs and non-coding RNAs, as well as proteins, cytokines, or growth factors, to target cells.^254^

### Cytoskeleton

The cytoskeleton, consisting of actin, microtubules and intermediate threads, is an advanced structural and complex network that is highly integrated and coordinated.^255,256^ Two cytoskeletal polymer systems are present in platelets: microtubules and microfilaments.^257^ Microtubules are non-membranous tubular structures arranged in a ring around platelets, maintaining platelets in a discoid shape, also known as the marginal band, with 3–24 layers, each with a diameter of about 25 nm.^258,259^ The main component of microtubules is tubulin.^260^ When platelets are activated, or microtubules are broken down by low temperatures or β1-microtubule protein knockdown, platelets become rounded.^6,261,262^ Microfilaments are fine filamentous structures that are generally not visible in platelets in the resting state. Microfilaments contain mainly actin filaments, about 5 nm in diameter, and a small number of short thick filaments of myosin. During platelet activation, actin is the driver of shape change, spreading and platelet contraction. When platelets are activated, many microfilaments appear in the cell matrix.^263–266^ In the narrow region between the microtubules and the plasma membrane, there is a thick layer of microfilament meshwork containing special microfilament structures called submembrane filament, which consists of actin.^267,268^ It has been shown that integrin α_IIb_β_3_ on the plasma membrane contact with the actin of submembrane filament, and when platelets are activated, actin extends from submembrane filament to the central part of platelets.^267^

## Role of platelets in physiological condition

### Hemostasis

**Fig. 5:**
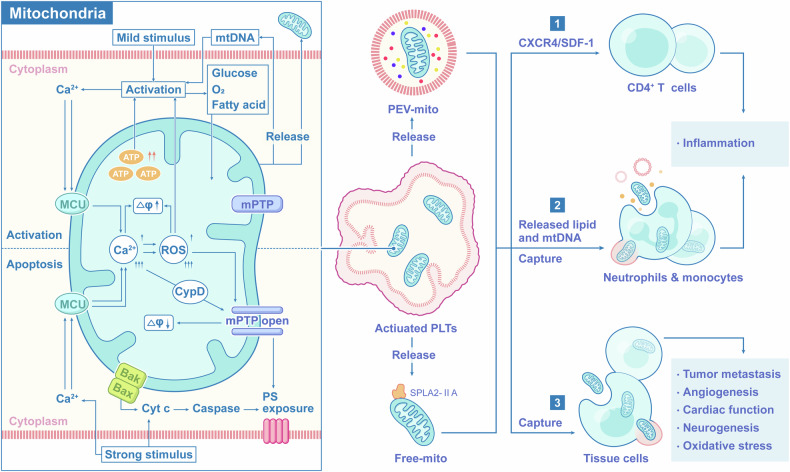
Biological functions of platelet mitochondria. In addition to providing energy to platelets through OXPHOS, platelet mitochondria are involved in regulating platelet activation and apoptosis. External stimuli can mediate intracellular calcium influx, leading to subsequent changes in mitochondrial membrane potential and increased OXPHOS. Under different intensities of stimulation, ROS and ATP produced by mitochondria promote platelet activation on one hand, and regulate platelet apoptosis through the CypD-mPTP pathway and apoptotic protein cascade pathway on the other hand. In addition, activated platelets can regulate immune response and various physiological functions by releasing mitochondria and mtDNA, which are captured by immune cells or tissue cells

As is well known, the most principal role of platelets is to be involved in hemostasis. Hemostasis is a vital physiological mechanism that halts bleeding at sites of vascular injury and preserves the integrity of blood vessels. The interplay between platelets, coagulation factors, and endothelial cells plays a pivotal role in driving this intricate process. To accomplish this, platelets rapidly adhere to the vessel wall as they circulate through the bloodstream and swiftly respond when blood vessels are damaged.^269^ This reaction is usually divided into several stages: (1) platelets adhesion to the wounded vessel wall, (2) platelet activation, and (3) the formation of platelet aggregates, aiming to form blood clots and seal the gaps formed in a vessel wall,^270^ i.e., primary hemostasis. This is additionally accompanied by activation of an enzymatic cascade reaction leading to fibrin deposition, i.e., secondary hemostasis^271^ (Fig. 5).

In physiological conditions, antiplatelet molecules released by endothelial cells, such as nitric oxide (NO) and prostacyclin (PGI_2_), inhibit interactions between platelets and endothelial cells.^272^ NO activates the production of soluble guanylate cyclase and cGMP, and platelet aggregation is subsequently prevented through phosphorylation of different targets by activated cGMP-dependent protein kinase (PKG).^273^ Most platelets circulate in a stationary disc-shaped form in the bloodstream and do not interact with healthy blood vessel walls. Seconds after vascular injury, vascular spasms occur and cause vasoconstriction, ultimately leading to cessation of blood flow. Both stationary (such as collagen) and mobile (such as thrombin, ADP, and TXA_2_) platelet agonists gather locally, promoting platelet adhesion to the subendothelial extracellular matrix through specific receptors.^274^ At low shear rates (100–1000/s), typical of venous systems or inside the atria, platelets can directly bind to extracellular matrix components such as collagen, laminin, and fibronectin. In regions with high shear stress (1000–4000/s), subendothelial von Willebrand factor (vWF) binds to the GPIbα receptor, triggering its unfolding and exposing several binding sites on the GPIb-IX-V complex. This facilitates the further attachment of GPVI to collagen and fibronectin within the subendothelial layer. Moreover, proteins such as fibrinogen, fibronectin, vitronectin, and vWF released from platelet granules strengthen platelet adhesion to the vascular wall by bridging platelet GPIIb/IIIa receptors with endothelial α_V_β_3_ integrins or intercellular adhesion molecule (ICAM).^275^ In addition to biglycan, other ECM components, such as fibrinogen and fibronectin, also play crucial roles in platelet adhesion and activation. These proteins interact with platelet surface receptors, including integrins and GPVI, thereby promoting platelet aggregation and activation at sites of vascular injury. This interaction enhances the formation of a stable platelet plug essential for hemostasis.^276^

These interactions, between GPVI and collagen specifically, as well as thrombin, activate platelets to undergo increase of cytosolic calcium, massive shape and ultrastructural changes. Activated platelets undergo spreading and degranulation, releasing factors including ADP, 5-hydroxytryptamine (5-HT), thrombin, and TXA_2_, which activates platelets by interacting with specific receptors and triggering downstream signaling like the GPIIb/IIIa activation, activating more stable and irreversible platelet aggregation and clot contraction, promoting hemostasis.^277,278^ Activated platelets also release TSP-1, which binds with high affinity to platelet GPIV to promote platelet activation and regulate hemostasis in vivo by inhibiting cAMP signaling and decreasing platelet sensitivity to PGI_2_.^279^ The activated platelet surface exposes PS, further promoting coagulation.^280^ In addition to releasing various coagulation factors, activated platelets also release platelet microvesicles (PMVs) that contain a variety of proteins, including vWF, fibronectin, and vitronectin, as well as membrane receptors such as P-selectin, GPIIb/IIIa, and GPIV. These PMVs play a key role in further promoting thrombosis by enhancing platelet aggregation and supporting the formation of a stable clot at the site of vascular injury.^249^

### Thrombosis

Following the initial attachment of platelets to the vascular wall, the subsequent strong adhesion activates signaling cascades that induce a shift in platelet morphology, causing them to flatten from their initial spherical shape.^281^ As activated platelets within the thrombus continue to interact with circulating platelets, they facilitate further platelet aggregation through GPIIb/IIIa-mediated binding, promoting the formation of a dense platelet plug.^57^ Moreover, the production of thromboxane A2 (TXA2) via COX-1 not only activates platelets but also triggers a feedback loop that amplifies platelet aggregation.^282^ Fibrinogen is subsequently converted into fibrin through both endogenous and exogenous pathways. This conversion facilitates the recruitment of more platelets to the site of injury, while simultaneously constructing a fibrin meshwork.^283^ Thus, the final platelet thrombus is composed of a center formed by activated platelets and fibrin, and a shell composed of the secondary agonists adenosine diphosphate and thromboxane, as well as loosely accumulated platelets.^284^

## Role of platelets in pathological condition (Fig. 6)

### Role of platelets in immune response

Platelets, primarily known for their crucial role in hemostasis, have also been increasingly recognized as key regulators of inflammation and immune responses during infection. On the one hand, platelets participate in the immune response by direct recognition of pathogen-associated molecular patterns (PAMPs) and DAMPs.^76^ On the other hand, activated platelets release immune mediators to coordinate the efficient recruitment of granulocytes, monocytes and lymphocytes and form aggregates by acting as a ‘bridge’ between leukocyte recruitment and certain adhesion molecules (selectin and integrin) and chemokine-dependent events in the vascular endothelial cascade.^285^ In addition, platelets release granule-containing anti-microbial and cytotoxic proteins, ROS and DAMPs, and synthesize lipid mediators.^88^

#### Recognition and responses of pathogens

When pathogens enter the human body, platelets recognize and respond to these foreign invaders through a series of complex steps. Platelets recognize pathogens through their surface pattern recognition receptors, especially TLRs, and are activated.^286^ When activated, platelets secrete PF4 from AGs, which then binds to polyanions (P). This interaction induces a conformational shift in PF4, exposing novel epitopes that trigger the generation of anti-PF4/P antibodies. These antibodies, in turn, facilitate platelet-mediated bacterial clearance by promoting platelet recognition of PF4-coated bacteria. This bactericidal function necessitates the activation of FcγRIIA receptors, the proper functioning of GPIIb/IIIa integrins, and the preservation of the platelet cytoskeleton integrity.^287^ Platelets are capable of recognizing and responding to signals released from damaged tissues, such as collagen and TXA_2_, and respond to inflammation through endogenous activation pathways, including the activation of PI3K and PKC.^288^ These pathways activate platelets, leading to their morphological changes, increased adhesiveness, and the release of cytokines and chemokines (like PDGF, TNF-α, IL-1β, CXCL4 and CCL5) that attract and activate neutrophils, monocytes, and lymphocytes, promoting leukocyte circulation and migration to inflammatory sites through the upregulation of adhesion molecules and selectins, enhancing the immune response to pathogens.^289^ In some cases, platelets can even transport contained cytokines and signaling molecules directly to the inflammatory area in the form of microparticles, such as CXCL5, IL-1β, and P-selectin.^290–292^

#### Communications with immune cells

##### Neutrophils

In infection and inflammatory responses, platelet-immune cell complexes facilitate more efficient pathogen clearance.^293^ Neutrophils, a type of leukocyte within the polymorphonuclear class, play a crucial role in the human immune system and are mainly responsible for resisting the invasion of pathogens such as bacteria and fungi.^294^ Neutrophils are the first white blood cells to arrive at infection sites in innate immunity. Platelets recruit and enhance neutrophil activation and aggregation by releasing chemokines and cytokines (such as CXCL4, CXCL7, TGF-β) and binding to P-selectin glycoprotein ligand-1 (PSGL-1) on neutrophil surface.^295,296^ This interaction is particularly significant in the formation of platelet-neutrophil aggregates, which play a key role in inflammation and thrombus formation.^297,298^ In interactions with pathogens, activated platelets induce the formation of neutrophil extracellular traps (NETs), which are composed of DNA and antimicrobial proteins, capturing and killing pathogens, limiting the spread of infection, by releasing PF4 and high-mobility group box 1 protein (HMGB1).^299^ It has been reported recently that in the condition of sepsis, cGAS-STING signal was also activated in platelets to bind with STXBP2, inducing the SNARE-dependent secretion of platelet granules, leading to thrombosis and NETs formation through P-selectin.^300^

##### Monocytes

Monocytes are a type of leukocytes, playing a crucial role in immune response, inflammation regulation, and tissue repair.^301^ Platelets regulate inflammatory responses by interacting with monocytes through CD40L/CD40 and P-selectin/PSGL-1 and forming platelet-monocyte aggregates (PMA), which can serve as markers of platelet activation and monocyte inflammatory response and enhance the inflammatory response and phagocytic ability of monocytes, making them more effective in clearing pathogens and cellular debris.^302^ This interaction also activates monocytes, promoting them to secrete inflammatory cytokines like TNF-α and IL-1β, exacerbating inflammation.^303^ On the other hand, the interaction between platelets and monocytes can reduce inflammation by enhancing IL-10 production and decreasing TNF-α levels in monocytes.^304^ In addition, platelets also regulate the differentiation of monocytes. The expression of CD16 is increased by activated platelets, which induces monocytes to transit to an intermediate phenotype.^302^ The binding of platelet P-selectin to monocyte PSGL-1 drives platelet-monocyte interaction and forms synaptic connections, activating the cross presentation program of monocytes and differentiation into dendritic cells.^305^

##### T cells and B cells

During inflammation, except for innate immunity, platelets also collaborate with T cells and B cells to enhance the efficiency of immune responses. Platelets can regulate T cell proliferation and differentiation by releasing multiple cytokines and chemokines like PF4, CCL5, and CD40L.^306^ The interaction of platelets with T cells via CD40L can enhance T cell activation and proliferation, and regulate CD8^+^ T cell responses.^307,308^ PF4 is also associated with TGF-β to regulate the response of CD4^+^ T cells.^309^ P-selectin/PSGL-1 also exists between platelets and T cells, blocking the immunosuppressive function of Tregs.^310^ In addition, platelet-derived mitochondria can regulate CD4^+^ T cell activity through CXCR4/SDF-1.^153^ For B cells, platelet-recognizing antigens can stimulate B cells to produce antibodies and encapsulate pathogens.^287^ PF4 secreted by platelets increases the differentiation of hematopoietic progenitor cells into B-cell lineage through the activation of STAT5.^311^

##### Other immune cells

Besides, platelets can also regulate other immune cells through different factors and pathways, such as regulating macrophage polarization,^312,313^ regulating the toxicity of natural killer cells,^314,315^ and affecting the development and differentiation of plasmacytoid dendritic cells.^316^

#### PEVs in inflammation

In addition to platelets themselves, PEVs play a crucial role in the pathophysiology of the immune system by increasing their number or altering their granule content.^317^ PEVs, mainly including exosomes and microvesicles, are small membrane vesicles released through platelet agonist-mediated platelet activation in physiological states, and through inflammation and/or infection in pathological states.^318^ PEVs carry and deliver many bioactive molecules, including chemokines and cytokines, which participate in regulating immune responses through activation and recruitment of leukocytes.^319^ Normally, platelets are rarely found or absent in synovial fluid. However, under inflammatory conditions, PEVs enter the synovial fluid and have been identified in synovial fluid and are elevated in rheumatoid arthritis.^320^ These PEVs are pro-inflammatory, as they may can influence the pathogenesis of rheumatoid arthritis, promoting the migration and invasion of rheumatoid arthritis fibroblast-like synoviocytes by activating the NF-κB pathway mediated via CXCR2 signaling pathway.^290^ In a state of chronic inflammation, PEVs have the ability to exit the bloodstream and infiltrate the bone marrow microenvironment. Once inside, PEVs rapidly bind to bone marrow cells, including MKs and their progenitors (CD41^+^ cells), thereby altering the function and phenotype of MKs. In this way, PEVs can act as sentinels and messengers, conveying changes occurring in the plasma environment directly back to the cells in the bone marrow.^321,322^

#### Platelets and the complement system

The complement system is a part of the innate immune system. As a fast and effective immune monitoring system, it has different effects on healthy and altered host cells as well as foreign invaders.^323^ Evidence suggests that platelet activation and P-selectin expression can activate the complement system, characterized by increased C3b deposition, C3a generation, and C5b-9 formation.^324^ Platelet surface complement receptors bind to complements in plasma, promoting complement pathway activation, further enhancing platelet aggregation and activation, forming a positive feedback loop that amplifies platelets’ role in immune defenses.^325,326^

### Role of platelets in cancer

Extensive research has provided valuable insights into the multifaceted roles of platelets, particularly their interaction with tumorigenesis.^327–330^ Clinical evidence suggests that thrombocytosis (elevated platelet count) has been linked to an increased risk of cancer.^331^ Platelets play a crucial role in circulation, adhesion, infiltration, and survival as tumor cells proliferate and undergo circulatory dissemination.^332,333^

#### Tumors educate and activate platelets

Studies have shown that alterations in platelet RNA and protein profiles in cancer patients can influence various platelet functions beyond hemostasis. This unique phenotype group is called tumor-educated platelets.^334^ Tumor cells alter the function of platelets through released stimulatory factors, leading to significant changes in the platelet proteome and transcriptome, enhancing their pro-vascular growth, pro-metastatic, and pro-coagulant properties, which is called educating.^335,336^ The pro-tumor proliferative effect of platelets depends on the tight contact between platelets and cancer cells. This interaction exists both intravascularly (circulating tumor cells) and extravascularly (tumor cells in situ) with the aid of G protein-coupled receptor-mediated platelet extravasation into the tumor parenchyma and is dependent on the expression of ITAM-containing immune receptors such as GPVI, CLEC2, and FcγRIIa.^337–339^ In addition, thrombin and tissue factor, as well as other proteins and nucleic acids released by tumor cells, activate platelets through different pathways.^340–343^ Recent studies have shown that platelets also efficiently absorb EVs from invasive cancer cells, which transfer cancer biomarkers and activate platelets in CD63-dependent pathway, leading to thrombosis formation, which underscores the diagnostic value of platelet-related cancer biomarkers.^343^

#### Roles of platelets in tumor development

Activated platelets within the tumor microenvironment release various pro-survival, pro-angiogenic, and immunomodulatory factors that help establish and sustain both primary and metastatic tumors. Platelets can release granulocyte colony-stimulating factor (G-CSF), macrophage colony-stimulating factor (M-CSF), MMP9, and GM-CSF, increasing the release of bone marrow-derived cells into the circulation, which are recruited to the tumor microenvironment via platelet-derived VEGF, differentiate into mature endothelial cells, and induce the establishment of a tumor vascular network.^344^ As a result of neovascularization, in the tumor microenvironment, activated platelets secrete a variety of cytokines and growth factors, such as VEGF, CCL5, PDGF, TGF-β, PF4, and HGF. These substances facilitate tumor progression and metastasis by supporting angiogenesis, enhancing cell migration, and modulating immune responses, thus promoting both the growth and spread of the tumor.^345,346^ In addition, platelet miRNAs have regulatory functions on primary ectopic tumor growth rates and gene expression, including epithelial-mesenchymal transition (EMT)-related pathways, cell cycle, mitochondrial function, and sensitivity to chemotherapeutic agents.^347–349^ Thus, platelet-derived miRNAs have higher accuracy and specificity than traditional tumor detection markers and circulating miRNAs in tumor diagnosis.^350,351^

#### Roles of platelets in cancer metastasis

Platelets are involved in distant tumor metastasis mainly by promoting tumor EMT. Platelets activate the Wnt-β-catenin and NF-κB signaling pathways in tumor cells, enhancing EMT-related gene.^352^ Furthermore, it was shown that cancer cells acquire platelet mitochondria through the PINK1/Parkin-Mfn2 pathway to reprogram to a metastatic state. Platelet mitochondria regulate cancer cell GSH/GSSG ratio and ROS to promote lung metastasis of osteosarcoma.^157^ Tumor cells can also acquire mitochondrial-dependent function by absorbing mitochondria through PMVs, thereby stimulating an increase in cellular oxygen consumption and intracellular ATP levels, and exhibiting enhanced malignant features in migration and invasion.^353^ In addition to other factors, platelets in the tumor microenvironment also release lysophosphatidic acid, a lipid that mimics the signaling effects of growth factors. This compound stimulates the activity of multiple matrix metalloproteinases in cancer cells, aiding in the detachment of tumor cells from their primary location and promoting their invasion into the circulatory system.^354^ In addition, tumor cells rely on CD15 binding to P-selectin to adhere to platelets, and the adhered platelets enhance the proliferation and migration characteristics of tumor cells by increasing the adhesion between tumor cells and endothelial cells.^355^

#### Role of platelets in immune inhibition

Upon entering the bloodstream, tumor cells recruit and activate platelets, which induces further platelet activation and enhances aggregation, aiding in progressing the invasion-metastasis cascade by assisting tumor cells.^356^ Once cancer cells are in the bloodstream, platelets act as a key protective shield, safeguarding them from shear stress and immune system recognition. Tumor cell-expressed galectin-3 binds to platelet GPVI dimers, activates platelets, and forms a protective aggregation barrier around circulating tumor cells, protecting them from immune destruction.^357,358^ This phenomenon is known as tumor cell-induced platelet aggregation (TCIPA). Besides, tumor cells can evade immune surveillance by acquiring platelet-derived lipids, nucleic acids, and surface proteins through phagocytosis, uptake, and presentation.^359,360^ Platelets promote the recruitment and proliferation of cancer-associated fibroblasts (CAFs) through the release of PDGF, extracellular matrix deposition, and inhibit intra-tumor infiltration of immune cells through the TGF-β pathway.^361^ In addition, platelets regulate and inhibit the immunoreactivity and proliferative capacity of macrophages, NK cells, and T cells through released TGF-β, PDL1, serotonin, and PEG_2_, as well as surface P-selectin.^362–365^

### Platelets and other diseases

#### Platelets and thrombotic disorders

Thrombotic disease refers to a pathological state caused by abnormal blood clotting, which leads to the formation of blood clots and may cause a series of health problems. The main characteristic of thrombotic diseases is the formation of intravascular thrombi, which can block blood vessels, leading to insufficient blood supply and causing tissue damage and functional impairment, which is often attributed to the activation of the coagulation cascade or circulating platelets. Platelets are integral to the onset of numerous thrombotic conditions, such as atherosclerosis, arterial thrombosis, stroke, deep vein thrombosis (DVT), and pulmonary embolism.^366–370^

##### Atherosclerosis

Atherosclerosis is a chronic vascular disease, which is characterized by the gradual accumulation of lipids, cholesterol, calcium salts and other substances in the intima of the arteries, forming plaques (atherosclerotic plaques), resulting in narrowing and hardening of blood vessels, thus affecting blood flow.^371^ This disease is one of the main causes of cardiovascular diseases, including heart disease, stroke, and peripheral arterial disease.^372^

Two key pathophysiological mechanisms underpinning atherosclerosis are heightened platelet activation and elevated concentrations of low-density lipoproteins (LDL).^373^ Evidence indicates that platelets are involved not only in atherothrombotic complications but also in the early processes of atherogenesis.^374^ Platelet attachment to aberrant vWF on undamaged endothelium takes place at the early stages of atherosclerosis.^375^ Under pathological conditions, immobilized endothelial chemokine CXCL16, expressed in human atherosclerotic plaques, captures platelets from flowing blood, promoting CXCR6-dependent platelet adhesion to the human vessel wall, endothelial cells, and vWF, leading to irreversible platelet aggregation and an increase in intra-platelet calcium levels.^376^ Platelet-derived MMP-2 is also essential in the initiation of atherogenesis by activating endothelial PAR1, triggering endothelial p38/MAPK signaling, and inducing the expression of adhesion molecules like P-selectin.^377^ P-selectin plays an important role in atherosclerosis. Research shows that p-selectin knockout mice have less atherosclerotic lesions in the aorta.^378^ P-selectin-knockdown mice receiving wild-type platelets transplantation have 30% more lesions than those receiving P-selectin deficient platelets transplantation and are more prone to calcification.^379^

After adhesion, platelet activation occurs through multiple pathways, playing a significant role in both atherogenesis and atherothrombosis.^380^ The pivotal platelet collagen receptor GPVI/FcRγ-chain complex critically contributes to platelet activation through the ITAM pathway.^381^ Proprotein convertase subtilisin/kexin 9 activates platelets by directly binding to CD36, leading to the activation of Src, ERK5, and JNK, an increase in ROS generation, and activation of the p38/cPLA2/COX-1/TXA_2_ pathways.^382^ This activation triggers downstream pathways that induce platelet aggregation.^383^

After activation and aggregation, platelets release pro-inflammatory cytokines to promote the formation of atherosclerotic plaques.^384^ Platelets adhered to inflamed endothelium present CXCR6 to CXCL16-positive peripheral blood mononuclear cells (PBMCs), mediating an increased adhesion of PBMCs to the atherosclerosis-prone vessel wall and promoting the progression of atherosclerosis.^385^ Platelet-neutrophil interactions increase the release of Mrp8/14, inhibit neutrophil apoptosis through P-selectin, and upregulate the TLR4/myeloid differentiation factor 88/NF-κB pathway, which is a crucial inflammatory signaling pathway in atherosclerosis pathogenesis.^386^ Oxidized low-density lipoprotein (oxLDL)-activated platelets, via scavenger receptors SR-A and CD36, form PMA with proinflammatory monocytes.^302^ This process is accompanied by enhanced adhesiveness and thrombus formation. In contrast, activated platelets facilitate the oxidation of LDL particles via ROS production from NADPH oxidase, which is known to stimulate platelet activation^387^ and can be mediated by p47phox.^388^ Furthermore, Platelets also play a role in atherogenesis by promoting the uptake of ox-LDL cholesterol into the arterial walls, which results in the formation of foam cells.^389^ Targeting these pathways with small molecule drugs can reduce the incidence of cardiovascular events.^390–392^

##### Myocardial infarction (MI)

MI is an acute disease caused by sudden interruption of blood flow to the coronary artery, resulting in partial myocardial hypoxia and necrosis, often associated with atherosclerosis. Platelets contribute to the pathogenesis by triggering intense inflammatory responses.^393^ In acute ischemia/reperfusion injury, hypoxia induces extensive mitophagy in platelets in a FUNDC1-dependent manner and regulates platelet activation through mitochondrial quality control.^394^ In MI patients, TLR4 of platelets is activated, potentially in response to circulating LPS.^395^ MI leads to platelet internalization, resulting in the release of miR-223-3p, causing a reduction in the protective effect of acidic phosphatidylcholine on myocardial cells against ferroptosis.^396^ Lee et al.^397^ demonstrated that inhibiting coagulation receptor PAR4 on platelets can reduce coronary atherosclerosis and myocardial fibrosis, reduce leukocyte and platelet accumulation in atherosclerotic coronary arteries, providing a potential new therapeutic approach.

##### Stroke

Stroke is one of the leading causes of death and disability worldwide. Experimental and clinical evidence indicates that ischemic stroke frequently progresses due to a thromboinflammatory process, with both platelets and T cells playing key roles.^398^ CD84, part of the signaling lymphocyte activation molecule family, functions as a homophilic cell adhesion molecule and is prominently expressed on both immune cells and platelets. When released by platelets, CD84 interacts with CD4^+^ T-cells, promoting their motility and aggravating the expansion of infarcts after cerebral ischemia/reperfusion.^399^ Additionally, necrotic platelets engage with neutrophils, exacerbating brain damage after ischemic stroke, possibly through the mediation of CypD.^400^

##### Venous thromboembolism (VTE)

Venous thromboembolism encompasses DVT and pulmonary embolism. While platelets were not historically considered the primary factor in VTE, increasing experimental evidence suggests that they play an important role in the pathophysiology of VTE.^401^ A clinical trial found that elevated levels of platelet-neutrophil aggregates were associated with the risk of DVT occurrence, making it a potential marker for predicting DVT development.^402^ IL-9, a cytokine involved in many inflammatory diseases, facilitates platelet function through the JAK2/STAT3 pathway, thus promoting the development of DVT.^403^ Platelet-derived HMGB1, a key mediator of sterile inflammation, has been identified as a central regulator in the prothrombotic cascade involving platelets and myeloid leukocytes, promoting the formation of occlusive DVT.^404^ In cancer patients, podoplanin (PDPN), predominantly expressed in primary brain tumors, contributes to an increased risk of VTE by stimulating platelets through the platelet hem-ITAM signaling mechanism.^405^ CAFs are an important component of the tumor microenvironment, and CAFs and CAF-derived extracellular vesicles can induce CLEC-2-dependent platelet aggregation, exacerbating venous thrombosis.^406^ Furthermore, increased production of ROS with aging induces the activation of the mechanistic target of rapamycin complex 1 (mTORC1) signaling, enhancing platelet activation to promote aging-related VTE.^407^

#### Platelets and sepsis

Clinical data shows that sepsis caused by severe infections often accompanies thrombocytopenia,^408^ which is due to excessive platelet activation leading to large-scale thrombus formation, severe depletion of circulating platelet count, and resulting in platelet dysfunction.^409^ In animal experiments, LPS stimulation resulted in excessive activation of platelets, leading to a significant increase in adhesion, aggregation, secretion, and diffusion on fibrinogen, as well as the expression of platelet membrane glycoproteins. This was accompanied by a significant decrease in cGMP levels and abnormal distribution of platelet AGs, which was mediated by the PI3K-AKt-GSK3β pathway.^410^ Besides, platelet mitochondrial damage and elevated intracellular ROS play a crucial role in promoting platelet aggregation through the LPS/TLR4 pathway, which involves phosphorylation of AKT, PKC, and p38.^411^ A study on 18 sepsis patients found that during the initial stage of sepsis-induced platelet mitochondrial uncoupling, there is a soluble plasma factor that does not inhibit the electron transport system. Mitochondrial uncoupling accompanied by gradual and significant increase in respiratory capacity.^412^ However, persistent mitochondrial dysfunction and respiratory chain enzyme inhibition will further affect platelet reactivity to exogenous agonists, as well as impaired aggregation and secretion.^413^ In addition, immune receptors such as GPVI and CLEC-2 also play important roles.^414^ Clinical trials have indicated that GPVI dysfunction in sepsis occurs prior to a significant drop in platelet count. In sepsis, platelets are unable to effectively transduce signals through GPVI, resulting in the failure of tyrosine phosphorylation of Syk or LAT. Consequently, a deficiency in GPVI signal transduction can serve as an early diagnostic indicator for sepsis.^415^ The renin-angiotensin system (RAS) is elevated in sepsis patients, and the elevation of AngII promotes oxidative stress through AngII type 1 receptor (AT1R) dependent pathway, directly stimulating platelet apoptosis.^416^ In addition, the plasma levels of soluble TREM like transcriptose-1 derived from platelets are significantly elevated in sepsis patients, and it can act as an endogenous DAMP that activates monocytes through TLR4/MD2 complex, followed by sustained immunosuppression.^417^ A recent study has shown that cyclic guanosine monophosphate adenosine monophosphate (cGAMP) derived from sepsis promotes the binding of platelet STING to STXBP2, assembly of SNARE complexes, granule secretion, and subsequent sepsis thrombosis.^300^

#### Platelets and diabetes

Diabetes mellitus is a metabolic condition marked by elevated blood glucose levels, with the most significant pathological feature being microangiopathy. This is driven by oxidative stress and the excessive generation of reactive oxygen species during hyperglycemia, which plays a central role in the development of various diabetic complications, including cardiovascular diseases, nephropathy, and retinopathy.^418,419^ Due to the interaction between thrombosis and inflammation in diabetes, the involvement of platelets in this has received much attention.^420^

In diabetes, the imbalance between circulating coagulation factors and the endothelial cell surface leads to a hypercoagulable state, primarily driven by platelet hyperactivity.^421^ Hyperglycemia, insulin resistance, and the resulting increase in oxidative stress lead to endothelial dysfunction, whose triggered infiltration of inflammatory cells and the formation of an inflammatory milieu trigger the formation and rupture of atherosclerotic plaques and further activation of platelets.^422^ Platelet surface receptors GPIIb/IIIa and P-selectin are activated in high osmotic state.^423^ Meanwhile, the hyperglycemic environment can also lead to PKC-dependent platelet activation.^424^ In addition, platelet function is directly controlled by insulin through functional insulin receptors (IR) present on the surface of platelets.^425^ Platelets undergo drastic physiological and morphological changes during activation, which requires glycolysis and oxidative phosphorylation to produce more energy.^426^ Therefore, mitochondria may become potential regulatory factors for platelet function and potential targets for antiplatelet therapy. Prolonged exposure of platelets to elevated glucose concentrations can result in alterations to platelet mitochondrial function, including heightened oxygen consumption and an increase in mitochondrial membrane potential,^427^ causing excessive production of ROS, and exacerbates platelet activation.^428^ Moreover, Parkin is involved in mitochondrial quality control through the process of mitophagy and is significantly expressed in platelets, both in healthy individuals and those with diabetes. It is thought to modulate platelet aggregation and activation by interacting with integrin signaling pathways and proteins such as FREMT3, PDIA, and ILK. Consequently, platelet Parkin may play a regulatory role in mitophagy and platelet activation in diabetes, presenting itself as a promising therapeutic target.^429^ Metformin is the first-line drug for the treatment of type 2 diabetes and the only drug that has been proven to reduce cardiovascular complications in diabetes patients. Research has indicated that metformin inhibits mitochondrial complex I, thereby safeguarding mitochondrial function. This action reduces mitochondrial hyperpolarization, prevents the overload of reactive oxygen species, and mitigates membrane damage caused by activated platelets in diabetes. Additionally, metformin prevents the release of mtDNA, which helps in reducing the risk of thrombosis.^430^

Inappropriately activated platelets in diabetes synthesize PDGF, which exerts its cellular role by binding to tyrosine kinase transmembrane receptors,^431^ with multiple downstream signaling pathways affecting the course of diabetes and its complications. Briefly, hyperglycemia leads to endoplasmic reticulum stress and oxidative stress with subsequent triggering of inflammatory signals and platelet abnormal activation, and activation of downstream pathways of abnormally elevated PDGF leads to impaired insulin secretion and insulin resistance in a variety of cells.^432–434^ Platelet activation triggers the local activation of the coagulation cascade and the formation of a fibrin network that stabilizes the thrombus, ultimately resulting in vascular complications.^435^ Platelet bioenergy spectrum analysis revealed that in type 2 diabetes patients with coronary in-stent restenosis, platelets heavily relied on fatty acid oxidation. This led to complex III deficiency, causing reduced mitochondrial respiration, increased production of mitochondrial oxidants, and low mitochondrial ATP production efficiency. These changes were closely associated with the secretion of platelet AGs and DGs.^436^ In addition, PEVs play an important role as messengers, linking inflammation and thrombosis in diabetes patients.^437^

#### Platelets and immune-mediated inflammatory diseases (IMIDs)

Immune-mediated inflammatory diseases (IMIDs) such as systemic lupus erythematosus (SLE), rheumatoid arthritis and psoriasis have a very high prevalence in the population.^438^ Cardiovascular complications are prevalent in IMIDs, and platelet activation induced by complement factors and immune complexes has been identified as a major contributor to thrombosis and increased risk of cardiovascular disease.^15^

Activated platelets release various signals that modulate the immune response and inflammation. For instance, PEVs are present in the joints of patients with rheumatoid arthritis, where they contribute to disease progression by inducing a cytokine response in synovial fibroblasts through IL-1.^439^ Platelet-derived mitochondria and mtDNA also play a vital role in the proinflammatory responses. In SLE patients, anti-dsDNA antibodies in the blood induce strong platelet activation by enhancing P-selectin expression and significant morphological and ultrastructural changes, which are accompanied by mitochondrial depolarization and decreased ATP content, indicating energy depletion.^440^ Activated platelets may release mitochondria through the mitochondrial apoptosis pathway and the extrusion of mitochondrial DNA (mtDNA), which is the main source of self-antigens, leading to the onset of lupus.^441^ Released mitochondrial and mtDNA cause the production of anti-mtDNA antibodies. Forming of immune complexes with anti-mtDNA autoantibodies can lead to their deposition into tissues, causing inflammation and organ damage.^442^ Additionally, mtDNA, a prototypical DAMP molecule and a strong inducer of type I interferon (IFN) production, is taken up by cells and detected by pattern recognition receptors,^443^ activating cGAS pathway and IFN production, leading to pro-inflammatory response.^444^ Patients with giant cell vasculitis have extracellular mitochondrial markers, which impair their ability to regulate extracellular mitochondrial clearance and may lead to excessive inflammation and platelet activation.^445^ Furthermore, in IMIDs, activated platelets influence their phenotype through interactions with innate and adaptive immune cells, promoting inflammatory and autoimmune responses with higher circulating levels of their aggregates.^446^ In addition to mediating inflammatory responses that damage tissues and organs, activated platelets release platelet-derived growth factors and TGF-β, leading to the hyperproliferation of renal mesangial cells, which are involved in the development of glomerulonephritis.^447^

#### Platelets and fibrosis

Fibrosis is caused by an imbalance in the repair response of wounds and connective tissue, leading to excessive deposition of extracellular matrix. Multiple organs may undergo fibrosis, including the liver, kidneys, heart, and lungs. The core mechanism of fibrosis is the sustained abnormal activation of myofibroblasts mediated by various signals. Activated platelets, as an important source of growth factors such as TGF-β, PDGF, and FGF in plasma, play an important role in fibrotic diseases.^448^ Idiopathic pulmonary fibrosis is a disease that causes scar formation and destruction of lung tissue. Studies have found that inducing platelet depletion in a mouse model of pulmonary fibrosis leads to reduced pulmonary fibrosis and moderate inhibition of lung function.^449^ By inhibiting platelet activation, the infiltration of neutrophils caused by platelet and neutrophil adhesion mediated by CD40-CD40L interaction was reduced, which has the potential to treat pulmonary fibrosis.^450^ Liver fibrosis is a downstream scar effect associated with chronic liver injury, where platelets aggregate along collagen fibers and are subsequently activated, initiating an inflammatory cascade by producing cytokines such as PDGF, thereby driving fibrosis.^451^ Ischemia/reperfusion injury and the resulting inflammation in acute kidney injury can set off a cascade of events in the kidneys, driving the progression to chronic kidney disease and, eventually, fibrosis. In this process, platelet-derived THBS1 plays a key role in stimulating macrophages to differentiate into a highly proliferative M2 subtype, which contributes to renal fibrosis by secreting excessive extracellular matrix component.^452^ Myocardial fibrosis is a characteristic of most heart diseases and a key factor leading to heart failure and its progression. In necrotic myocardial tissue, platelets recognize DAMPs through pattern recognition receptors and activate,^453^ promoting myocardial fibrosis by releasing signaling factors such as TGF-β1,^454^ serotonin,^455^ and p-selectin.^456^

## Platelet targeted therapy

As mentioned above, activated platelets participate in the progression of various diseases by forming blood clots, releasing cytokines, and communicating with multiple cells. Therefore, studying platelet-targeted therapy has become an effective treatment strategy for platelet activation-related diseases. At present, various drugs specifically targeting platelets for the prevention or treatment of thrombosis have been developed and approved, such as COX-1, P2Y_12_, integrin α_IIb_β_3_, phosphodiesterase, and PARs inhibitors.^13^ However, traditional antiplatelet therapy mainly targets thrombosis, and its main limitation is an increased risk of bleeding. Ideal antiplatelet drugs should selectively inhibit thrombosis without interfering with the hemostatic mechanism. In this regard, downstream signaling molecules mediated by receptors in platelet activation have attracted widespread research interest.^14^ Therefore, this review focuses on novel antiplatelet methods targeting other platelet surface molecules and mitochondria and their downstream signal transduction, which is not only limited to thrombotic diseases but also associated with various platelet activation-related diseases.

### Surface molecules targeted therapy

#### GPVI targeted therapy

Inhibition of GPVI has been linked to a marked reduction in collagen-induced thrombosis, while the risk of bleeding remains unaffected.^457^ As a result, research into GPVI inhibitors or antibody-mediated depletion has emerged as a promising pharmacological strategy for developing effective and safe antiplatelet therapies. Among the currently reported anti-GPVI antibodies with potential therapeutic effects, Fab fragment-based antibodies are one of the main strategies. It has been proved through in vitro thrombosis model and calculation simulation that under the condition of arterial blood flow, blocking GPVI with Fab fragment (ACT017) can promote the effective decomposition of thrombus formed on collagen or human atherosclerotic plaque materials, and its effect increases with the increase of shear rate of vascular wall.^458^ Voors Pette and her team further showed in clinical trials that intravenous administration of ACT017 had no significant effect on bleeding time, and it did not result in changes to platelet count, platelet GPVI expression, or plasma levels.^459^ Blocking the binding site between GPVI and collagen is another antiplatelet strategy. Revacept, a protein created by combining a human Fc fragment with a dimer of the GPVI extracellular domain (GPVI-Fc), has been tested in clinical trials with doses of 80 and 160 mg in patients undergoing percutaneous coronary intervention. The results indicated that the 160 mg dose effectively reduces platelet aggregation caused by high collagen concentrations. However, this reduction did not correlate with a decrease in myocardial injury, and bleeding complications remained minimal, with only a few incidents reported.^460^ In a separate clinical trial, the safety, tolerability, and effectiveness of Revacept (40 or 120 mg) were evaluated in individuals with carotid artery stenosis, transient ischemic attack (TIA), transient blackout, or stroke. The results demonstrated that a 120 mg dose of Revacept was effective in lowering the risk of new ischemic lesions and adverse health outcomes, including stroke, mortality, myocardial infarction, coronary intervention, and bleeding.^461^ Snake venom protease has been shown to cleave GPVI and inhibit platelet activation. Recent studies on a snake venom protease, mutalysin II, have shown that it can block platelet aggregation induced by GPVI agonists through cleaving GPVI, without significantly affecting hemostasis.^462^ In addition, the nucleotide-binding oligomeric domain 2 (NOD2) antagonist GSK669 has been shown to target GPVI, inhibit platelet adhesion to collagen, and reduce collagen-induced phosphorylation of Src, Syk, PLCγ2, and Akt.^463^

#### GPIb-IX-V targeted therapy

Another target of platelet inhibition is the GPIb/IX/V, which binds to and induces vWF-related thrombosis.^464^ Antibodies, snake venom derivatives, and fusion proteins targeting GPIb receptors have been demonstrated to inhibit the interaction between platelets and vWF. Anfibatide, extracted from the venom of *Agkistrodon acutus*, acts as a GPIbα antagonist by binding competitively to the GPIbα subunit of the GPIb-IX-V complex, thus inhibiting the interaction with von Willebrand factor (vWF). Studies have demonstrated that Anfibatide can significantly decrease platelet adhesion and thrombus formation while ensuring minimal risk of bleeding.^465^ In a randomized, open-label Phase I clinical trial at a single center, Anfibatide successfully inhibited platelet aggregation induced by vWF, while leaving bleeding time and coagulation largely unaffected.^466^

#### P-selectin targeted therapy

After platelet activation, P-selectin is released from AGs and transferred to the platelet membrane, promoting platelet adhesion and aggregation by acting as a platelet molecular switch, and plays a key role in tumor proliferation and immune cell aggregation.^467^ Crizanlizumab binds to p-selectin, thereby blocking its interaction with PSGL-1, for the treatment of sickle cell disease, but its application in other platelet activation-related diseases has not been validated.^468^ Incalcumab, a recombinant monoclonal antibody directed against P-selectin, has demonstrated potential in reducing inflammation, thrombosis, and atherosclerosis. Clinical trials have indicated that it can mitigate myocardial injury in patients with non-ST segment elevation myocardial infarction after undergoing percutaneous coronary intervention.^469^ In addition to anti-thrombotic effects, knockout of platelet p-selectin in mice has been shown to reduce tumor metastasis.^470^

#### CLEC-2 targeted therapy

CLEC-2 is an immunoglobulin receptor expressed on the surface of platelets, and its only ligand in the human body, PDPN, has been identified and is highly expressed on tumor cells.^105^ Studies have indicated that selectively inhibiting platelet CLEC-2 could provide an innovative approach to preventing hematogenous tumor metastasis and reducing cancer-associated thromboembolism.^471^ The use of 5-nitrobenzoate compound 2CP to specifically inhibit PDPN/CLEC-2 interaction can suppress PDPN-mediated platelet activation and enhance the chemotherapy efficacy of cisplatin.^472^

#### CD40L targeted therapy

Platelets and immune cells communicate and regulate immune responses through CD40/CD40L interactions. Antibodies that target the CD40-CD40L pathway hold significant promise for treating autoimmune conditions like rheumatoid arthritis, systemic lupus erythematosus, lupus nephritis, and inflammatory bowel disease.^473^ In a sepsis mouse model established by cecal ligation and puncture, treatment with CD40L-CD40-TRAF6 signaling pathway blocker compound 6877002 can alleviate intestinal barrier dysfunction, increase ZO-1 and occludin expression, and improve the survival rate of sepsis mice.^474^

### Signal transduction targeted therapy

#### Rho GTPases

Rho GTPases, along with their upstream regulators and downstream effectors, are essential in platelet activation and aggregation, positioning them as promising targets for the development of antiplatelet therapies.^475^ Currently, one of the most effective strategies for inhibiting Rho GTPase activation involves the use of small molecule inhibitors, such as Rhosin,^476^ which target the GEF binding surface on Rho GTPase to prevent its activation, inhibiting platelet activation, ROS production, platelet diffusion, secretion, and aggregation.^477^ In addition, downstream effectors of Rho GTPase, such as ROCK, are also excellent targets. HA-1077 is the only clinically approved ROCK inhibitor used for the treatment of cerebral vasospasm and pulmonary arterial hypertension.^478^ NSC23766 specifically inhibits Rac1 activation in a dose-dependent manner, blocking the secretion and aggregation of DGs.^479^ For another member of the Rho GTPases family, cdc42, its inhibitor CASIN blocks the formation of pseudopodia, secretion, and aggregation of platelets, as well as the phosphorylation of its downstream effector PAK.^480^ Another small molecule, Secramine, specifically inhibits the activation of Cdc42 in a RhoGDI-dependent manner and suppresses platelet adhesion, filamentous pseudopodia formation, and aggregation.^481^ These inhibitor studies indicate that pharmacological targeting of Rho GTPase through specific inhibitors can better determine their role in steady-state regulation and discover new antiplatelet drug.

#### PI3K

PI3K phosphorylates PIP2 to produce PIP3, which mainly participates in downstream signal transduction of GPCR and GPVI. Its dependent downstream targets are protein kinase A (PKA), PKG, and PKC.^482^ A recent study showed that the PI3Kβ inhibitor MIPS-9922 can effectively inhibit ADP-induced platelet aggregation, activation of integrin α_IIb_β_3_, and platelet adhesion to immobilized vWF, preventing arterial thrombosis without causing prolonged or excessive bleeding.^483^ Compared with clopidogrel combined with aspirin, PI3Kβ inhibitor AZD6482 combined with aspirin has a greater overall antiplatelet effect, but the likelihood of bleeding is significantly reduced.^484^

#### Syk-Btk axis

Collagen binds to GPVI to activate SRC kinase-dependent ITAM tyrosine phosphorylation and tyrosine kinase Syk activation.^485^ The active metabolite of the Syk inhibitor Fostamatinib (R406) can slightly inhibit the platelet response induced by atherosclerotic plaque homogenate.^486^ In addition, Syk downstream protein Btk inhibitors ibrutinib, acalabrutinib, ONO/GS-4059, BGB-3111 and Evobrutinib can inhibit platelet thrombosis in flowing blood on atherosclerotic plaque, but retain the hemostatic function of platelets.^470,487^

#### 12-LOX

12-LOX uses arachidonic acid released from membrane phospholipids as a substrate and oxidizes it into 12(S)-hydroxyeicosatetraenoic acid (12-HETE). The latter can amplify platelet activation mediated by GPVI and PAR4. Pharmacological inhibition of 12-LOX leads to reduced platelet aggregation, selective inhibition of the secretion of DGs and AGs, and inhibition of platelet adhesion of PAR4 and collagen under flowing conditions.^488^ ML355 is a selective 12-LOX inhibitor that can inhibit human platelet aggregation and the production of 12-LOX oxygen lipids.^489^

#### Non-canonical non-genomic morphogen signaling

In recent years, some components of morphogen pathways have been discovered in platelets, namely Notch, Sonic Hedgehog (Shh), and Wnt, which regulate platelet function and affect their survival through non-canonical, non-genomic signaling pathways in the context of thrombosis.^490^ The binding of Notch1 and its ligand Delta-like ligand (DLL)−4 can promote thrombus formation at the site of vascular injury by promoting the binding of integrin receptors to high-affinity fibrinogen, secreting dense and alpha particle contents, and platelet-leukocyte interactions.^491^ The non-canonical Shh signaling pathway is mediated by RhoA/AMPK, which can amplify agonist-driven platelet activation and arterial thrombosis formation.^492^ Therefore, targeting Shh signaling in platelets could serve as an effective antithrombotic strategy. Additionally, exogenous Wnt3a has been shown to negatively regulate RhoA-GTP signaling by disrupting the interaction between Dvl and Daam1,^490^ thereby inhibiting agonist induced platelet response.^493^

#### Nuclear receptor (NR) signaling

NRs represent mammalian protein families related to transcriptional regulation of human tissues, such as androgen receptors (ARs),^494^ estrogen receptor (ER),^495^ corticosteroid receptor (GR),^496^ regulating cell proliferation, differentiation, metabolism, and homeostasis, with the ability to trigger two different responses, genomic and nongenomic.^497^ Because platelets lack cell nuclei, several NRs present in human platelets can trigger non-genomic effects by physically interacting with cofactors and binding partners. These interactions lead to the initiation of rapid signaling events,^498^ regulating platelet-activated granule secretion and leading to the release of a range of chemokines, cytokines, growth factors, and anti-inflammatory factors, resulting in the progression of many diseases. This process is independent of transcriptional regulation and responds rapidly.^439,499^ Recently, several natural and synthetic ligands of these NRs have been found to influence platelet function by engaging various mechanisms. The reduction of platelet adhesion and thrombus formation under collagen flow treated with glucocorticoid receptor ligand prednisone may be mediated by modulating signaling events downstream of the P2Y_12_ receptor.^500^ Liver X receptor ligands GW3965 and T0901317, as well as natural ligands 27-OH cholesterol and 24-(S)- hydroxycholesterol, can inhibit the reactivity of agonists by converting platelets into procoagulant platelets. This seems to be achieved through mechanisms such as dysregulation of intracellular calcium signaling, depolarization of mitochondrial membrane potential independent of CypD, and production of ROS.^501^ After treatment with bile acid receptor ligand GW4064, significant reduction in thrombus stability was also observed.^502^ In addition, retinoic acid receptors can regulate protein synthesis in human platelets by binding to mRNA subsets and blocking translation. Platelets treated with their ligands showed significantly altered levels of protein synthesis.^503^

### Mitochondria-targeted therapy

Mitochondria are an efficient energy source required to maintain the rapid response of platelets to atherosclerotic plaque, and their functions play an important role in the process of platelet activation and apoptosis. Therefore, targeted regulation of platelet mitochondrial function has become an effective treatment strategy for platelet activation-related diseases.^504^ Mitochondria regulate platelet activation by switching between OXPHOS and glycolysis. Inhibition of mitochondrial pyruvate dehydrogenase kinase (PDK) activity by dichloroacetic acid (DCA) shifts metabolism from aerobic glycolysis back to OXPHOS, effectively inhibiting platelet aggregation and glucose uptake.^505^ Dimeric pyruvate kinase M2 (PKM2) is a key regulatory factor in aerobic glycolysis. The use of small molecule inhibitor ML265 to restrict the formation of PKM2 dimers can effectively inhibit platelet activation, aggregation, clot contraction, and in vitro arterial shear stress-induced platelet thrombosis in humans and mice.^506^ The β-oxidation of fatty acids maintains ATP levels in stimulated platelets, which may become an attractive therapeutic target for novel antiplatelet drugs.^129^ Platelet activation during ischemia/reperfusion is closely linked to the generation of ROS. Studies have demonstrated that vitamin C can neutralize ROS, decrease cytochrome c, Bax, and caspase-9 levels in mitochondria while increasing Bcl-2 expression. These actions work together to prevent platelet apoptosis and reduce platelet aggregation via the mitochondrial pathway.^507^ The mitochondrial mechanism of the antiplatelet effect of hydroquinone derivatives is related to the inhibition of ROS and ATP production. Studies have shown that ortho-carbonyl bicyclic hydroquinone can induce a decrease in mitochondrial membrane potential and ATP levels, and significantly inhibit platelet aggregation.^508^ Compound 5-bromo-1-(2-chloro-3,6-dihydroxy-4,5-dimethylphenyl) pentan-1-one exhibits antiplatelet activity by reducing mitochondrial energy production.^509^ MPTP inhibition is another potential target, and cyclosporine A inhibits CypD, thereby reducing the Ca^2+^ sensitivity of PTP opening and decreasing the formation of procoagulant platelets.^510^ In addition, TMEM16F is a Ca^2+^-dependent phospholipid cascade, and platelet procoagulant activity can be directly blocked by inhibiting TMEM16F. However, the inhibition of TMEM16F may be related to bleeding, and there are currently few reports on TMEM16F inhibitors.^511^

## Discussion and perspectives

### Omics analysis of platelets

Platelets were initially discovered and studied as the main components responsible for hemostasis and promoting thrombosis in the blood. However, with the continuous deep research on platelet structure and physiological functions, the current understanding of platelets has expanded to include many common pathological processes and disease progressions. Understanding the biological mechanisms and molecular pathways through which platelets play a role in these pathological processes is helpful in developing targeted treatment strategies for platelets.^512,513^

In recent years, advanced omics technologies such as genomics, transcriptomics, proteomics, and metabolomics have provided more comprehensive insights into the biological diversity of platelets in different cell-type-specific functional demands and physio-pathological regulations.^514^ Focusing on proteomic studies of peptides and matched protein subsets in platelets in different status and different populations such as obese and old people can quantify disease-specific platelet biomarkers, providing important research tools for identifying new diagnostics and drug targets for personalized and precision medicine.^515^ Poscablo et al.,^516^ through bulk and single-cell transcriptomics, identified an aging-enriched population of megakaryocyte progenitor cells (MkPs) with unique molecular characteristics. Compared to normal MkPs, these aging-enriched MkPs exhibit enhanced proliferative capacity and reconstruct circulating platelet supply through age-induced cellular mechanisms, producing platelets with higher reactivity and thrombogenicity. Rohlfing et al.,^517^ through metabolomic and lipidomic analyses, conducted in-depth research on heme-induced platelet activation and thrombosis, demonstrating that this process is regulated by the sGC-cGMP-cGKI signaling axis. Targeting platelet cGMP levels may therefore be a new strategy for controlling thrombosis formation in patients with hemolytic crises and severe limb ischemia. Since biological processes are complex and holistic, single omics data alone are insufficient to systematically and comprehensively elucidate the molecular regulatory mechanisms of complex physiological processes. Multi-omics analysis can simultaneously study biological problems from both “cause” and “effect” perspectives and validate their relevance. A study analyzed clinical phenotypes, transcriptomes, proteomes, metabolomes, and immune systems from blood cells or plasma samples of patients with severe acute respiratory syndrome coronavirus 2 (SARS-CoV-2) Omicron infection. Through integrated multi-omics analysis, enhanced interferon-mediated antiviral characteristics of platelets were detected, with platelets preferentially forming extensive aggregates with leukocytes to regulate immune cell function.^518^ Multi-omics analysis provides new methods and approaches for platelet research, promoting a deeper understanding of platelet function and disease mechanisms. In the future, further optimization of integrated multi-omics technologies and analysis methods is needed to improve data quality and interpretation accuracy, explore the correlation between platelet multi-omics data and clinical manifestations, and construct disease prediction models and personalized treatment plans.

### Platelet biomarkers of diseases

In addition to platelets themselves, platelet biomarkers, as important indicators for early diagnosis and monitoring of diseases, have received increasing attention in recent years. D’Ambrosi et al.^519^ investigated the synergistic effect of circRNA and mRNA signals derived from platelets as biomarkers for detecting lung cancer, providing potential combination diagnostic features for lung cancer detection. Three snoRNAs (SNORA58, SNORA68, and SNORD93) were found to be significantly upregulated in tumor-educated platelets (TEPs) of esophageal cancer (ESCA) patients, serving as non-invasive biomarkers for ESCA diagnosis and early detection.^520^ These biomarkers not only reflect the activity and functional status of platelets but also unveil molecular mechanisms associated with disease occurrence and development. Further exploration is needed in the future to elucidate the mechanisms of action of novel platelet biomarkers in the early diagnosis and monitoring of various diseases and to develop more sensitive and specific detection techniques, such as high-throughput sequencing and mass spectrometry analysis, to enhance the sensitivity and accuracy of platelet biomarker detection. Additionally, integrating platelet biomarkers with other biomarkers to construct multi-parameter disease diagnostic models will also improve diagnostic accuracy and reliability.

### Platelet-targeted gene therapy

Platelet-targeted gene therapy is an emerging therapeutic strategy aimed at using genetic engineering techniques to modulate the function and activity of platelets, enabling sustained expression of new proteins in vivo to treat specific diseases.^521–523^ Hemophilia A (HA) is an X-linked genetic disorder caused by a deficiency of FVIII. Targeting FVIII expression to platelets using 2bF8 lentiviral genes enables the continuous release of platelet-derived FVIII, effectively treating HA through bone marrow transplantation or transgenic platelet transfusion.^524,525^ However, challenges remain regarding the potential development of immune reactions to transgenic products or vectors. In the future, it is necessary to further explore novel platelet-targeted genes, such as miRNA and CRISPR-Cas9-mediated gene editing technologies, and their mechanisms of action in treating different diseases. Developing more precise and efficient gene delivery systems, such as viral vectors and nanoparticles, to enhance the targeting and transfection efficiency of gene therapy, is also crucial. These efforts will provide new avenues and methods for the further development and application of platelet-targeted gene therapy in clinical practice, bringing new hope and possibilities for disease treatment.

### Artificial intelligence (AI)-designed platelet-targeted drugs

Identifying target compounds plays a key role in the development of drugs aimed at platelets. To optimize and accelerate the process of predicting potential candidates, various machine learning models have been introduced. One such model, the Keras MLP, was utilized to scan a chemical library for possible inhibitors of platelet-derived growth factor receptor β (PDGFRβ).^526^ The deep learning algorithm developed by Luo et al., namely RNGS + DNN, identified a compound, wedelolactone, from the FDA approved drug library that differs structurally from conventional drugs, and demonstrated potential therapeutic effects on thrombocytopeni.^527^ Liu et al. used feedback system control (FSC) to combine AI calculations with bench experiments to screen and combine representative compounds derived from herbs with antiplatelet activity and combined them with dual antiplatelet therapy (DAPT) to study the optimization of combination drugs, which can help design personal antiplatelet therapy strategies.^528^ Another research shows that the combination of AI assisted discovery of characteristics of single nucleotide polymorphisms (SNP) and clinical parameters is likely to be used to develop race specific precise drugs for antiplatelet therapy in patients with diabetes peripheral arterial disease.^529^ Moreover, leveraging AI for dynamic prediction could revolutionize the risk stratification of ischemic or bleeding events following DAPT with drug-eluting stent implantation. This approach has the potential to offer personalized decision-making support, enhancing the management of DAPT treatment.^530^ In short, the rapid development of AI will provide technical support for the development of platelet-targeted drugs, as well as drug safety and personalization.

## Conclusion

In conclusion, platelets are not only crucial for hemostasis but also play a vital role in a wide range of physiological and pathological processes. The continuous expansion of knowledge in platelet biology holds significant therapeutic implications, offering new avenues for treating various diseases. Sustained research and innovation in platelet-targeted therapies, coupled with a deeper understanding of their molecular mechanisms, will pave the way for novel and more effective treatment approaches, ultimately improving patient outcomes and advancing medical science.

**Fig. 6:**
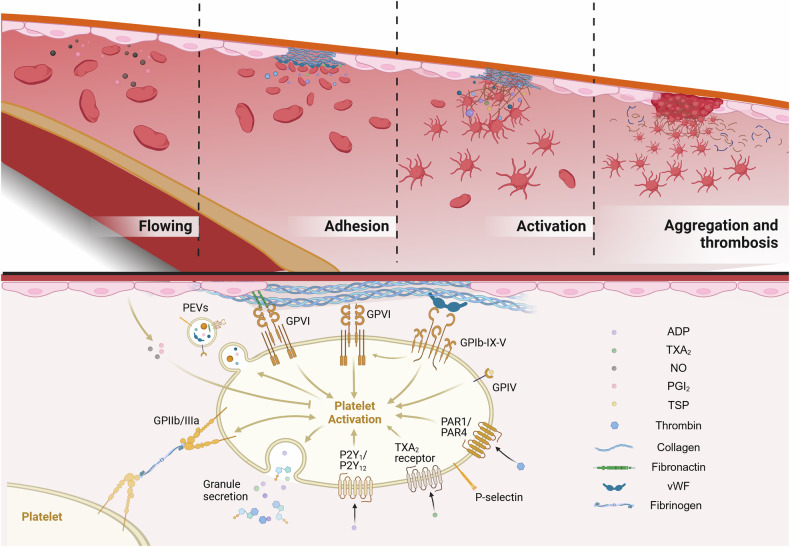
Roles of platelets in the biological processes of hemostasis and thrombosis. Under physiological conditions, platelets are regulated by the release of NO and PGI_2_ from endothelial cells to maintain resting state and flow in circulation. When blood vessels are damaged, agonists and adhesion proteins quickly accumulate and promote platelet adhesion to the subendothelial extracellular matrix through surface receptors. After adhesion, platelets are activated by agonists and undergo morphological changes and degranulation. The released cytokines bind to platelet surface-specific receptors and further activate platelets through downstream signaling, recruiting free platelets in the circulation to aggregate. Ultimately, the fibrin network and platelets jointly form the thrombus

**Fig. 7:**
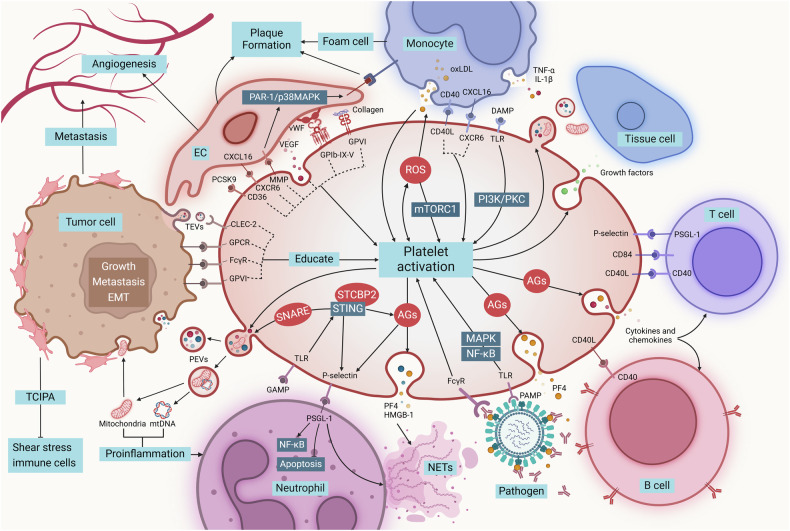
Role of platelets in pathological conditions. This figure depicts the intercellular communication and signaling pathways through which platelets are widely involved in various pathological processes and disease development. Regulating immune response and inflammation is an important mechanism for platelets to participate in diseases, including recognizing antigens through TLRs, secreting pro-inflammatory factors, recruiting and activating immune cells, and secreting vesicles and mitochondria. Inflammatory reaction is the key pathological process of thrombotic diseases, complications of diabetes and autoimmune diseases. In addition, platelets promote tumor progression by promoting tumor cell growth and metastasis, EMT, endothelial cell angiogenesis, and forming TCIPA to suppress immune surveillance
